# Cost-effectiveness of FIT and a FIT-based model to optimise symptomatic diagnosis of colorectal cancer: health economic modelling for the COLOFIT project

**DOI:** 10.1136/bmjph-2024-002089

**Published:** 2025-06-24

**Authors:** Chloe Thomas, Olena Mandrik, Jim Chilcott, Colin Crooks, David Humes, Willie Hamilton, Colin Rees, On behalf of the COLOFIT research group, Colin Rees

**Affiliations:** 1 Sheffield Centre for Health and Related Research, The University of Sheffield, Sheffield, UK; 2 Gastrointestinal and Liver Theme, National Institute for Health Research (NIHR) Nottingham Biomedical Research Centre, University of Nottingham, Nottingham, UK; 3 Translational Medical Sciences, University of Nottingham, Nottingham, UK; 4 Nottingham Colorectal Service, Nottingham University Hospitals NHS Trust, Nottingham, UK; 5 University of Exeter Medical School, University of Exeter, Exeter, UK; 6 Population Health Sciences Institute, Newcastle University, Newcastle upon Tyne, UK; 7 Department of Gastroenterology, South Tyneside and Sunderland NHS Foundation Trust, South Shields, UK

**Keywords:** Risk Assessment, Risk Assessment, economics, statistics and numerical data

## Abstract

**Introduction:**

Fecal immunochemical testing (FIT) at a threshold of 10 mg haemaglobin (Hb)/g is used in English primary care to prioritise urgent referral for colorectal cancer (CRC) investigation in symptomatic patients. The COLOFIT algorithm, based on FIT score, age, sex and blood results, performs better than FIT alone for identifying CRC. We assessed the cost-effectiveness of COLOFIT compared with FIT and investigated optimal risk thresholds.

**Methods:**

An individual patient-level simulation model was developed, with synthetic populations constructed from data used to validate COLOFIT. Referral criteria based on different FIT scores and COLOFIT-assessed risk thresholds were modelled using probabilistic and scenario analyses. Outcomes included costs, quality-adjusted life years (QALYs) and cost-effectiveness measured using incremental net monetary benefit (INMB) based on a willingness to pay threshold of £20 000/QALY.

**Results:**

COLOFIT at a CRC risk threshold of 0.64% has a 98% probability of being more cost-effective than FIT 10 mg Hb/g (INMB is £5.67 per person), while detecting similar numbers of cancers. Cost-effectiveness is achieved by cost savings from reducing referrals outweighing QALYs lost through reorienting expedited CRC diagnoses from younger (<50) to older (≥70) patients. Cost-effectiveness improves as risk thresholds rise. High structural uncertainty around cancer progression during diagnostic delay and diagnosis of other serious bowel diseases considerably affects cost-effectiveness.

**Conclusions:**

COLOFIT is likely to be more cost-effective than FIT alone and could help alleviate pressure on diagnostic services. However, strategies to improve diagnosis in the under 50s would be necessary to mitigate potential harm. Further research should assess how COLOFIT impacts cancer survival and diagnosis of other serious bowel diseases.

**WHAT IS ALREADY KNOWN ON THIS TOPIC**

- Fecal immunochemical test (FIT)-based triage for urgent referral of patients presenting with colorectal cancer (CRC) symptoms is current practice in the UK, but diagnostic ability could potentially be improved using a newly developed algorithm that incorporates FIT, age, sex and full blood count results (COLOFIT).

**WHAT THIS STUDY ADDS**

- Using the COLOFIT algorithm instead of FIT is likely to be cost-effective as it enables costly unnecessary referrals to be reduced, while diagnosing equivalent numbers of CRC.
- COLOFIT reorients expedited CRC diagnoses from people aged <50 to those aged ≥70, meaning it is likely to result in a small amount of quality-adjusted life year loss despite its cost-effectiveness.

**HOW THIS STUDY MIGHT AFFECT RESEARCH, PRACTICE OR POLICY**

- Adoption of COLOFIT would enable scarce diagnostic resources to be better allocated in a cost-effective and cost-saving way, but strategies should be put in place to improve diagnosis in people aged <50 to mitigate potential harm in this age group.
- Pilot studies are recommended to enable the validity of assumptions around COLOFIT performance, cost and ease of implementation to be assessed ahead of any national roll-out.

## Introduction

 Colorectal cancer (CRC) accounts for 10% of all cancer deaths in the UK.^1^ Despite the existence of a national screening programme, 90% of cases are diagnosed following the development of symptoms, presenting at a later stage with poorer outcomes.^2^ CRC symptoms are ubiquitous and frequently non-specific, and while referral guidelines prioritise high-risk patients for colonoscopy through urgent suspected cancer (USC) referral pathways (previously called ‘two week wait’ referrals),^3^ around 19% of diagnoses are made following non-urgent general practitioner (GP) referral pathways,^2^ with patients often waiting many weeks before receiving a diagnostic investigation. A similar percentage of patients present with an emergency admission following a complication of their cancer.^2^ There is evidence that such diagnostic delay can result in poorer survival for CRC patients.^4^ However, increasing the number of urgent referrals to mitigate this problem is not currently practical in the UK and many healthcare systems given the limited capacity for colonoscopy and other investigative procedures such as CT colonography (CTC).^5^ Furthermore, these constraints upon endoscopy services have been exacerbated in England by the extension of the CRC screening programme to younger age groups.^6^ Sparing people unnecessary investigation is also important given that colonoscopy and other definitive investigations may be uncomfortable for patients and have small risks of serious harm.^7^ There is a need to reduce unnecessary referrals while simultaneously improving identification of patients likely to have CRC to ensure that available diagnostic resources are used in the most effective and efficient way.

Lower gastrointestinal (GI) symptoms are poor predictors of CRC.^8^ Symptomatic diagnosis of CRC has been facilitated by the introduction of fecal immunochemical testing (FIT) in primary care, as FIT is highly sensitive for CRC.^9^,^12^ For those presenting with lower GI symptoms, a FIT threshold of ≥10 mg haemoglobin (Hb)/g feces is currently recommended to determine who should be offered referral.^13^ However, given its recent introduction, there is currently no data about the performance of FIT over the long term. Furthermore, the number of false positives for CRC at this threshold is relatively high and is increasing as FIT is used more widely, including in individuals who would never previously have been suspected to have CRC, for example, due to young age. It is therefore necessary to examine whether the performance of FIT for detecting CRC can be improved by including other personal or clinical characteristics into the risk assessment, and to determine the optimal risk threshold for referral to maximise efficient use of existing colonoscopy and CTC capacity.

The COLOFIT study is a programme of work that included developing and validating a multivariable algorithm to establish the optimal use of FIT for identifying CRC risk in patients presenting to the GP with symptoms of possible CRC. A review of existing risk prediction models indicated that FIT-based models generally performed much better than those without FIT, but that few existing models had been rigorously tested through internal and external validation.^14^ The CRC risk algorithms developed as part of the COLOFIT study were based on data from a large population in Nottingham and included age, sex, FIT and haematological parameters within a full blood count as variables within either a Cox proportional hazards model or a logistic regression model.^15^ In derivation and validation populations, both models were found to perform similarly and significantly better than FIT alone in predicting risk, in particular by reducing the number of false positive results.^15 16^ In this study, we present a health economic modelling analysis that assesses the potential cost-effectiveness of the COLOFIT algorithm compared with FIT alone in England and investigates the optimal threshold for usage in a population presenting to the GP with symptoms of possible CRC.

## Materials and methods

A health economic model was developed in R software (V.4.2.1),^17^ to evaluate the cost-effectiveness of the CRC risk algorithm developed in the COLOFIT project (hereafter referred to as COLOFIT) compared with using FIT alone, at a range of different thresholds of CRC risk. The study takes the perspective of the National Health Service (NHS) in England.

**Figure 1:**
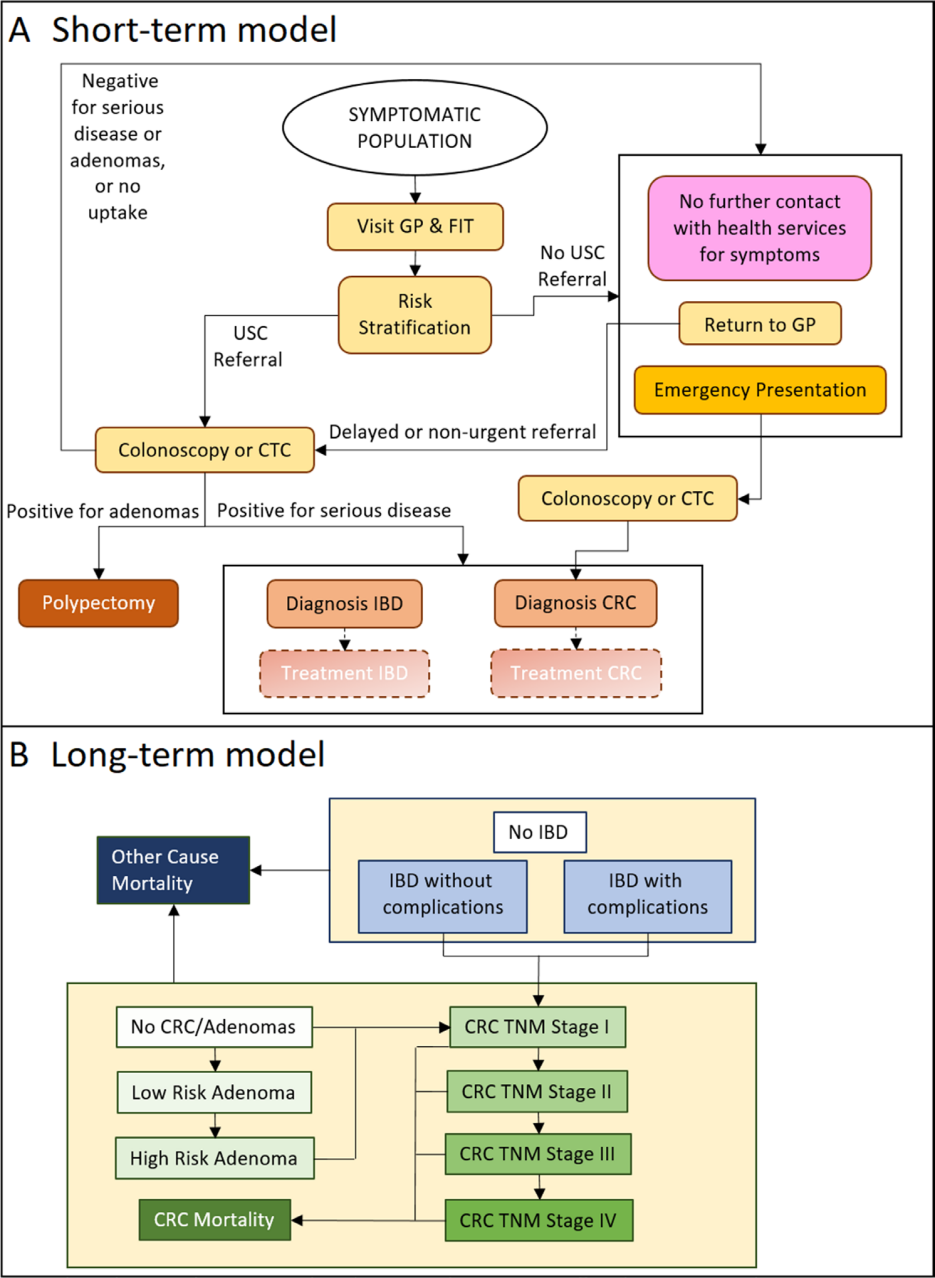
Model diagram showing the structures of the short-term diagnostic model and the long-term Markov style model. Boxes in the short-term model represent events, and boxes in the long-term model represent health states. CRC, colorectal cancer; CTC, CT colonography; FIT, fecal immunochemical testing; GP, general practitioner; IBD, inflammatory bowel disease; TNM, tumour, node, metastases; USC, urgent suspected cancer.

The model is an individual patient-level simulation with a short-term model that simulates the process and outcomes of initial diagnosis, and a long-term Markov style model with annual cycles and a lifetime horizon, which ensures that all potential long-term impacts of disease on survival, costs and benefits are included (figure 1). Conceptual modelling defined the model structure and scope prior to model build. While the aim of the project was CRC detection, conceptual modelling identified the importance of including other serious bowel diseases in the model, in particular high-risk adenomas and inflammatory bowel disease (IBD), as both of these conditions benefit from early detection and can be diagnosed via FIT positivity, and colonoscopy or CTC, and IBD presents with similar symptoms,^11 18 19^ meaning that COLOFIT introduction is likely to impact on the diagnosis of some cases. However, the ability of COLOFIT to detect these conditions is untested, making its performance compared with FIT alone unknown. As a compromise, both conditions were included in the constructed model described below, but the impact of diagnosing them early was ‘switched off’ for the main analyses and only included as part of additional scenario analysis (see below).

The model population at baseline represents adult patients who had attended their GP with symptoms of possible CRC and had then performed a FIT upon the request of the GP. Synthetic populations were constructed for model purposes based on multivariate sampling of aggregate data from the Nottingham populations that were used to derive or to validate COLOFIT,^15^ with the validation population being used in the base case analysis. Patients in the synthetic population had a set of correlated personal characteristics, including demographic information such as age and sex, and test results such as FIT and blood tests. Distributions of most variables, particularly FIT, are highly skewed. Data were transformed as necessary and the generated values were refitted within quantile thresholds, enabling distributions to be recreated in the population. A full description of how the populations were constructed and their summary characteristics can be found in the online supplemental technical methods document.

Each individual was assigned at baseline an underlying mutually exclusive health state with respect to CRC (which included CRC stages I–IV), low-risk or high-risk adenoma, or normal epithelium. IBD was included as a separate health state. CRC risk was estimated using either the Cox proportional hazards or logistic COLOFIT algorithms,^15^ with the Cox algorithm being used in the base case analysis. CRC status was then applied randomly to individuals at baseline based upon this risk. The modelled prevalence of CRC (1.2% in the validation population, 1.5% in the derivation population) closely reproduced the 1-year incidence in the Nottingham data. Information about the prevalence of adenomas and IBD was limited/unavailable from the Nottingham data, so an alternative published source was used to inform this, based on the known relationships of these conditions with FIT and age.^20^ It was assumed that COLOFIT would have some sensitivity for high-risk adenomas, as FIT does, so this health state was allocated based on COLOFIT score, but with different levels of prevalence in individuals with FIT >10 and FIT <10 as suggested by Nottingham data. Low-risk adenomas were allocated randomly in the population as there is evidence that the prevalence is similar in general and USC populations.^20 21^ IBD was allocated based on age and FIT score.

The short-term model incorporated costs of the initial GP appointment and FIT test in all individuals. Risk stratification was carried out either using the FIT score directly or risk as predicted by the COLOFIT algorithm. In the COLOFIT arm of the model, costs of doing a full blood count in people who did not already have one were also included (£8.38 per person for phlebotomy plus haematology).^22^ In addition, a small cost for implementing and running the algorithm was included, assumed to be £0.01 per person based on annuitisation of an estimated up-front capital cost (personal communication COLOFIT team). In the Nottingham population, 91% of people referred to secondary care with a FIT already had a full blood count within a year of the FIT of 14 days following it, so additional blood test costs were only included for the remaining 9% in the base case analysis. The flexibility of the individual patient-level nature of the model enabled any threshold of CRC risk predicted either by FIT value alone or through COLOFIT to be chosen. Individuals with scores equal to or above the selected threshold were referred through the USC pathway, in which case CRC (and adenomas/IBD if modelled) was assumed to be detected immediately dependent upon uptake and colonoscopy/CTC sensitivity for those conditions. Those below the threshold were not referred via the USC pathway, in which case CRC (and IBD if included) diagnosis was assumed to be delayed. This was also the case for anyone who chose not to take up USC referral, or whose condition was missed by colonoscopy/CTC.

CRC diagnosis in people not diagnosed through USC was assumed to be delayed by a mean of 3 months based on evidence from a UK study,^4^ with each patient having a personalised delay ranging from 2 weeks to 2 years. IBD diagnostic delays were assumed to be 1.34 years, based on data for time to diagnosis in people who are not diagnosed within 6 months of symptom onset.^19^ USC diagnosis was assumed to incur costs and harms of colonoscopy/CTC,^722^,^25^ while delayed diagnosis additionally incurred costs of extra GP appointments and emergency presentations in a proportion of delayed cases.^2 26^ Delayed diagnosis was assumed to result in a stage shift for CRC calculated through the lifetime Markov model (and higher incidence of complications for IBD^18 19^), leading to differences in costs, quality of life and survival that projected into the long term.

The lifetime Markov model was based upon our previously published CRC screening model,^27^,^29^ incorporating transition probabilities relating to CRC development, progression and diagnosis. CRC stage progression parameters in our screening model represent mean progression of both subclinical and clinical (symptomatic) CRC, but it is possible that progression could be faster in people with symptoms than represented by these parameters. This was tested in sensitivity analysis. CRC mortality was based on survival data by stage, age, sex and time since diagnosis for all CRC cases in England,^30^ as detailed survival data by route to diagnosis were not available. As only 11% of cases are detected through screening against 58% of cases detected through primary care referral and 24% through emergency presentation,^31^ it seems likely that the data are reasonably representative of stage-specific survival in the symptomatic population who have attended their GP. Other cause mortality was based on National Life Tables.^32^ All model parameters are described fully in the online supplemental technical methods document.

An independent researcher not involved directly in model code development (OM) checked the code for errors. A set of validations was carried out to test the performance of COLOFIT and FIT in the synthetic populations and compare against the Nottingham data. This indicated that the performance of FIT and COLOFIT could be replicated reasonably well in the model, although tended to diverge as risk thresholds increased (online supplemental technical methods document).

Model outcomes included costs, life years (LYs), quality-adjusted life years (QALYs), CRC mortality, number of USC referrals and number of CRCs diagnosed at USC. Cost-effectiveness was assessed through calculating incremental net monetary benefit (INMB), based on valuation of a QALY at £20 000. Results were extracted across the whole population per million patients and for subgroups defined by age and sex. Costs were measured in £UK at 2023 values. All costs and QALYs were discounted at 3.5% in the base case analysis in line with National Institute of Health and Care Excellence (NICE) guidelines.^33^

Model base case analyses compared a wide range of FIT and COLOFIT thresholds against referring all people to USC, with results compared against all other results in incremental analysis. The primary analysis compared the current NICE-recommended FIT threshold of ≥10 µg/mg (FIT 10) against a risk threshold of ≥0.64% as estimated by COLOFIT (COLOFIT 0.64%), as these are approximately equivalent in the number of CRCs detected at USC. Other important thresholds assessed related to 1%, 2% and 3% levels of risk as identified by the COLOFIT algorithm study (COLOFIT 1% vs FIT 13; COLOFIT 2% vs FIT 28, and COLOFIT 3% vs FIT 40).^15^ Due to some differences between the real population used in that study and the synthetic population developed for the model, these threshold comparisons do not represent perfectly equivalent risk. Results at multiple thresholds were therefore graphed to enable comparisons of outcomes across different levels of CRC risk.

In addition to the comparison of different thresholds, a set of scenario analyses was performed to investigate key structural uncertainties in the model. These included:

1. Incorporating detection of adenomas and/or IBD in the model.
2. Using a synthetic population based on the Nottingham derivation cohort instead of the validation cohort.
3. Using the COLOFIT logistic model to estimate risk rather than the Cox model;
4. Adding an additional harm of colonoscopy equivalent to loss of a full day of QALYs in everyone who undergoes one, to represent the patient burden of undergoing the procedure.
5. Increasing CRC stage shift caused by diagnostic delay through either maximising annual CRC transition probabilities and/or doubling diagnostic delay to a mean of 6 months.
6. Using higher (5%) or lower (1.5%) discount rates.
7. Increasing the costs of COLOFIT by assuming that all patients would incur additional costs of blood tests rather than just 9% as observed in Nottingham.

All model analyses were initially performed deterministically based on two million patients, with the primary and 1%, 2% and 3% risk threshold analyses for FIT and COLOFIT also performed using probabilistic sensitivity analysis based on 500 model runs of 100 000 patients each.

Patients and the public were involved in the reporting and dissemination plans of our research. A reporting checklist is provided as online supplemental material.

## Results

**Figure 2:**
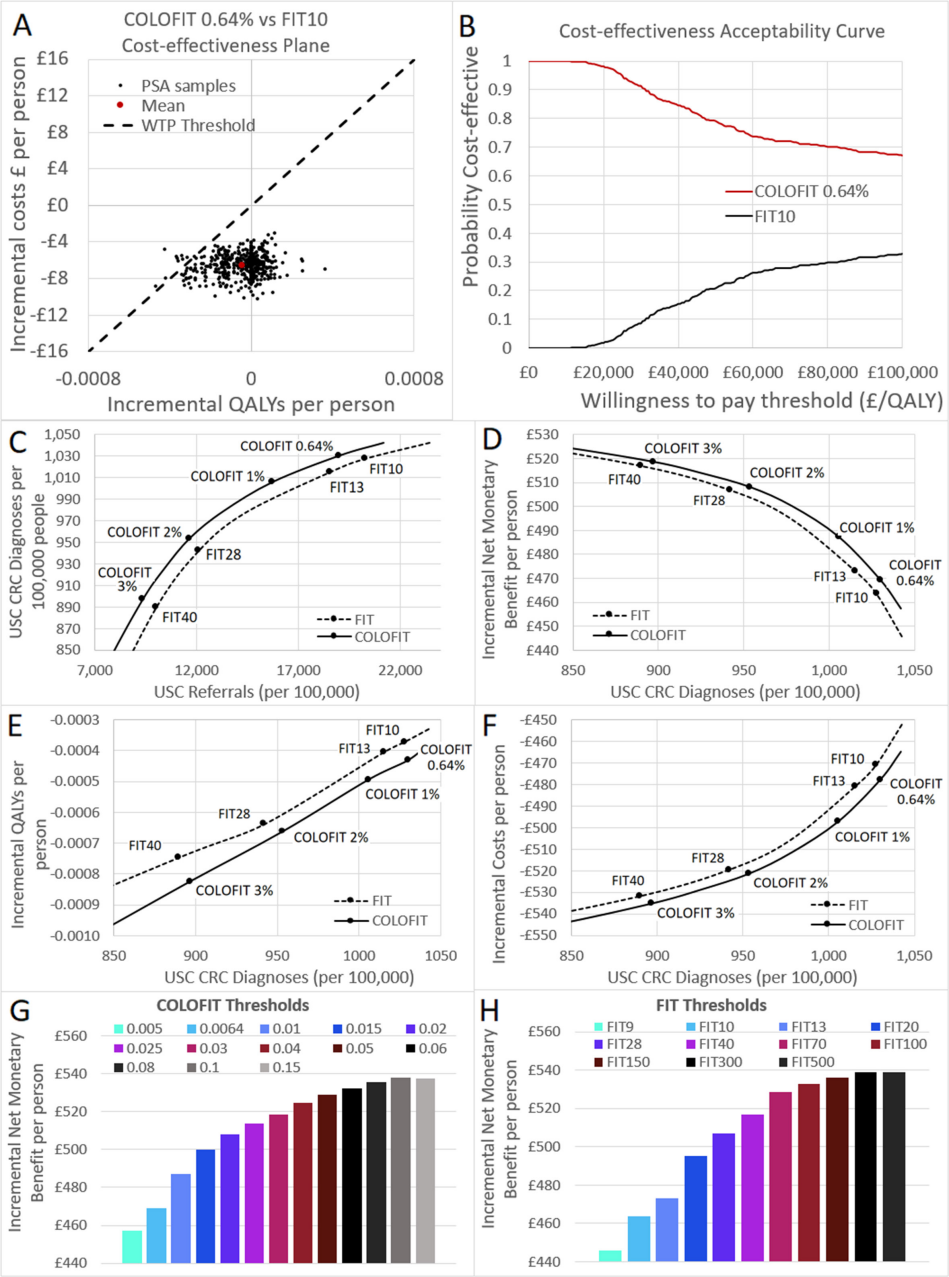
Base case results comparing FIT and COLOFIT at different thresholds. (**A**) Distribution of PSA results on the cost-effectiveness plane for the FIT10:COLOFIT 0.64% comparison. (**B**) Cost-effectiveness acceptability curve indicating probability cost-effective at different WTP thresholds for the FIT10:COLOFIT 0.64% comparison. Plots of (**C**) USC referrals; (**D**) incremental net monetary benefit; (**E**) QALYs and (**F**) costs against USC CRC diagnoses for different thresholds of FIT and COLOFIT compared with sending all urgently. Graphs indicating net monetary benefit at different thresholds of (**G**) COLOFIT and (**H**) FIT compared with sending all urgently. CRC, colorectal cancer; FIT, fecal immunochemical testing; PSA, probabilistic sensitivity analysis; QALYs, quality-adjusted life years; USC, urgent suspected cancer; WTP, willingness to pay.

The results of the primary probabilistic sensitivity analysis comparing FIT 10 and COLOFIT 0.64% suggest that COLOFIT would be more cost-effective than FIT with an INMB of £5.7 m per million patients and 98% probability of being the most cost-effective option (table 1, figure 2). There is a small reduction in incremental QALYs (−45 per million patients) and LYs (−53 per million patients) compared with FIT, and 1.5 additional CRC deaths are expected per million people despite higher numbers of USC pathway diagnoses (27 additional USC pathway diagnoses per million). However, the negative QALYs are overwhelmed by the incremental cost savings made of £6.6 m per million patients due to lower numbers of USC referrals required (12 505 fewer per million). Plots of INMB at different FIT and COLOFIT thresholds indicate that COLOFIT is more cost-effective than FIT at diagnosing the same numbers of USC CRC, although the INMB between FIT and COLOFIT diminishes and becomes negligible as thresholds increase (figure 2), and the probability that COLOFIT is more cost-effective than FIT diminishes from the 2% risk threshold upwards (table 1).

**Table 1:** Results from probabilistic sensitivity analysis comparing USC referral based on different FIT and COLOFIT thresholds against referring all people urgently, and two-way comparisons

Strategy(compared with sending all to USC)	Incremental NMB	Incremental costs	Incremental QALYs	Incremental life years	Incremental USC CRC diagnoses	Incremental CRC deaths	Total USC referrals	Probability more cost-effective than comparator
	Per 1 000 000 people							
FIT 10	£460 m(£334 m; £601 m)	−£469 m(−£612 m; −£345 m)	−425(−1029; −329)	−593(−1433; −18)	−1386(−2555; −674)	31(−20; 100)	202 428(199 904; 204 893)	100%
FIT 13	£469 m(£341 m; £613 m)	−£479 m(−£626 m; −£353 m)	−461(−1150; −50)	−643(−1583; −32)	−1509(−2751; −750)	34(−20; 110)	185 823(183 145; 188 030)	100%
FIT 28	£503 m(£365 m; £659 m)	−£517 m(−£675 m; −£380 m)	−701(−1621; −147)	−974(−2192; −159)	−2311(−4091; −1230)	50(−20; 140)	120 530(118 557; 122 285)	100%
FIT 40	£513 m(£372 m; £672 m)	−£529 m(−£691 m; −£389 m)	−835(−1828; −234)	−1163(−2529; −277)	−2837(−4765; −1540)	59(−10; 160)	99 778(98 014; 101 340)	100%
COLOFIT 0.64%	£466 m(£339 m; £611 m)	−£475 m(−£622 m; −£350 m)	−470(−1115; −53)	−646(−1539; −39)	−1359(−2477; −655)	32(−20; 110)	189 923(187 430; 192 091)	100%
COLOFIT 1%	£483 m(£351 m; £633 m)	−£494 m(−£646 m; −£364 m)	−561(−1629; −70)	−769(−1822; −64)	−1623(−2921; −810)	38(−20; 110)	157 275(154 789; 159 355)	100%
COLOFIT 2%	£503 m(£365 m; £660 m)	−£518 m(−£678 m; −£381 m)	−755(−1600; −140)	−1035(−2167; −152)	−2201(−3852; −1185)	52(−20; 150)	116 519(114 450; 118 416)	100%
COLOFIT 3%	£514 m(£375 m; £674 m)	−£532 m(−£695 m; −£392 m)	−932(−1975; −221)	−1277(−2622; −286)	−2767(−4691; −1560)	63(−20; 170)	93 373(91 615; 95 006)	100%
**Comparison**	**Per 1 000 000 people**							
COLOFIT 0.64%versus FIT 10	£5.7 m(£0.65; £9.82)	−£6.6 m(−£8.9 m; −£4.3 m)	−45(−322; 113)	−53(−416; 176)	27(−70; 140)	1.5(−20; 20)	−12 505(−13 796; −11 205)	98%
COLOFIT 1%versus FIT 13	£13.8 m(£7.3 m; £20.0 m)	−£15.8 m(−£20.8 m; −£11.2 m)	−99(−413; 94)	−126(−545; 150)	−114(−250; 0)	5(−20; 30)	−28 148(−29 435; −26 950)	100%
COLOFIT 2%versus FIT 28	£0.4 m(−£5.4 m; £4.6 m)	−£1.5 m(−£3.0 m; −£0.3 m)	−54(−367; 185)	−61(−488; 285)	110(−40; 270)	2(−30; 40)	−4011(−5016; −3014)	61%
COLOFIT 3%versus FIT 40	£1 m(−£5.9 m; £5.8 m)	−£2.9 m(−£4.6 m; −£1.4 m)	−97(−488; 165)	−115(−648; 269)	70(−100; 260)	4(−30; 45)	−6405(−7345; −5395)	66%
COLOFIT 3%versus COLOFIT 2%	£10.2 m(£3.2 m; £15.1 m)	−£13.7 m(−£17.9 m; −£10 m)	−177(−531; 1)	−243(−711; 16)	−565(−920; −320)	11(−20; 50)	−23 146(−24 085; −22 210)	100%

Note that 11 943 USC CRC diagnoses are expected in this population if all people are referred urgently. 95% credible intervals are shown in parentheses.

COLOFIT algorithm developed for COLOFIT project; NMB net monetary benefit (calculated based on a threshold of £20 000 per QALY).

CRC, colorectal cancer; FIT, fecal immunochemical test; m, million; QALY, quality-adjusted life year; USC, urgent suspected cancer.

For either FIT or COLOFIT, cost-effectiveness compared with sending all patients to USC referral increases with higher thresholds, with probabilistic results indicating that COLOFIT 3% is considerably more cost-effective than either COLOFIT 0.64%, COLOFIT 1% or COLOFIT 2%. Deterministic results indicate that thresholds above 3% risk could be even more cost-effective than those below, with INMB peaking at COLOFIT 10%, or FIT 500 (figure 2). However, differences between FIT, COLOFIT and different thresholds are minimal at this level of risk, and it should be noted that the COLOFIT algorithm has not been validated above a 3% risk of CRC threshold.^15^ As with the FIT versus COLOFIT comparison, these results are driven by cost savings from fewer USC referrals, with LYs and QALYs being lost as thresholds increase due to higher numbers of CRC with delayed diagnosis.

**Figure 3:**
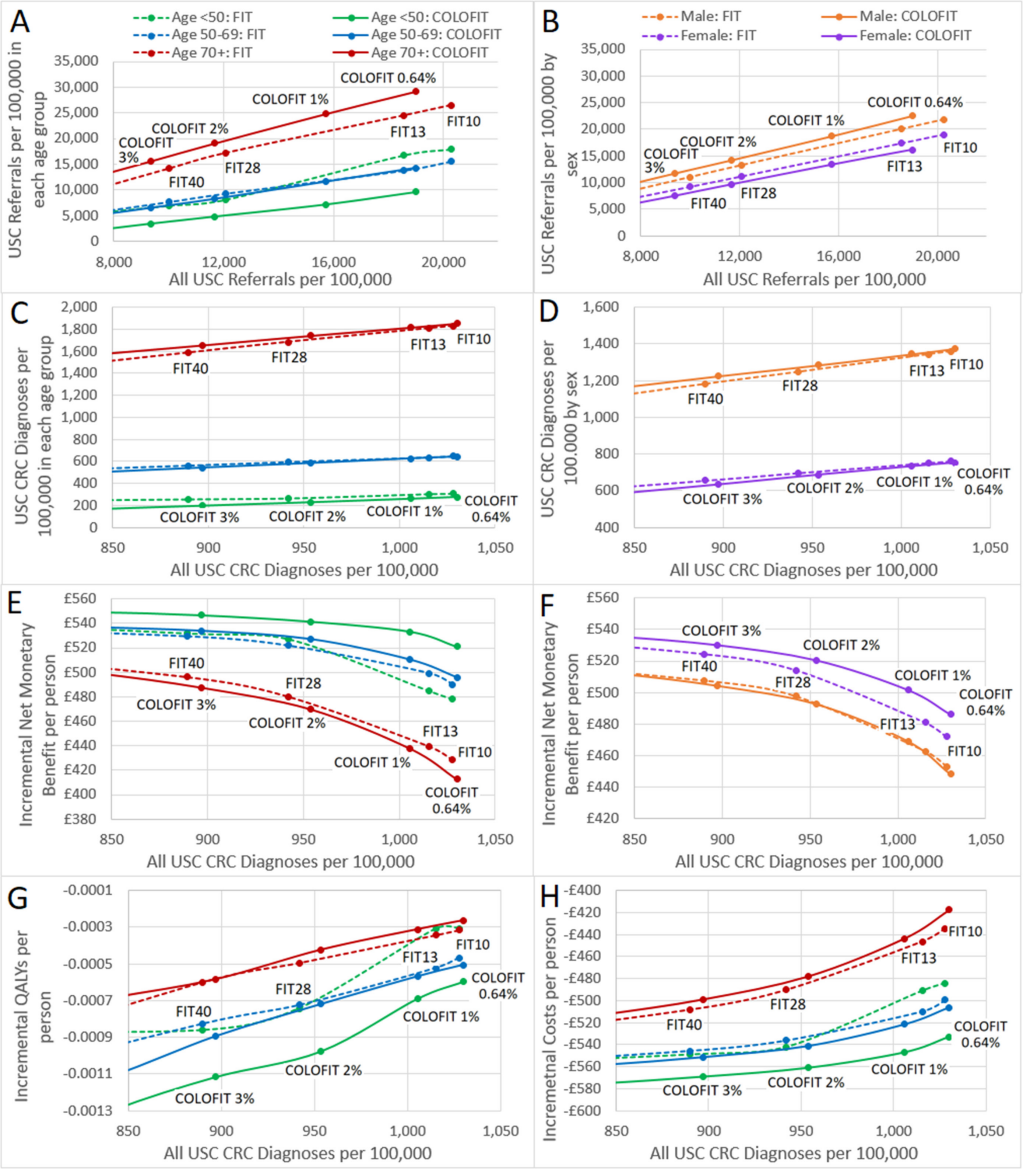
Plots showing FIT and COLOFIT at different thresholds compared against sending all urgently in subgroups defined by age and sex for (**A, B**) number of USC referrals plotted against all referrals; (**C, D**) number of USC CRC diagnoses; (**E, F**) incremental net monetary benefit; (**G**) QALYs and (**H**) costs. (**C–H**) All plotted against all USC CRC diagnoses. CRC, colorectal cancer; FIT, fecal immunochemical testing; QALYs, quality-adjusted life years; USC, urgent suspected cancer.

Subgroup analysis by age and sex indicates that COLOFIT reorients USC referrals from low yield young people and from women towards higher yield older people and men, which enables the algorithm to identify the same number or slightly more CRCs than FIT, with fewer USC referrals (figure 3 and online supplemental table S1). The reduction in USC referrals means that COLOFIT saves more costs and is more cost-effective overall compared with FIT, particularly in the young and in women. However, in reorienting USC referrals, COLOFIT also redirects expedited CRC diagnoses from the young to the old and from women to men. This results in LY and QALY loss in these groups, particularly for the young, who have more potential to benefit from early CRC diagnosis due to their longer life expectancy. Modelling suggests that 0.6–0.85 QALYs are lost for every delayed diagnosis in the under 50 age group compared with less than 0.2 QALYs lost for every delayed diagnosis in the over 70 age group (online supplemental table S2). This means that QALY loss from fewer CRCs diagnosed early in young people outweighs QALY gain from more CRC diagnosed early in older people, resulting in QALY loss overall across the population. Optimal thresholds for COLOFIT may be slightly lower in the young compared with the older age groups, but in all groups are above the 3% risk threshold (online supplemental figure S1). There is no clear difference in optimal threshold by sex.

Scenario analysis indicates that cost-effectiveness estimates vary widely if different assumptions are made around key structural model uncertainties (table 2, online supplemental table S2 and figure S2a, b). If adenomas and/or IBD are incorporated in the model, the optimal strategy is to refer everyone to USC, with cost-effectiveness reducing as thresholds increase, and COLOFIT being less cost-effective than FIT across thresholds. The reason for this is from much higher QALY loss, particularly in the young who are less likely to be referred through COLOFIT, but who are impacted disproportionately by adenomas and IBD being missed. In the case of IBD inclusion, no cost savings accrue despite fewer USC referrals due to the higher cost of treating cases with delayed diagnosis. Using a synthetic population based on the Nottingham derivation cohort produces results broadly similar to the validation cohort, providing FIT/COLOFIT comparisons are made based on similar numbers of USC CRC diagnoses (see online supplemental figure S2a, b for curves demonstrating this). Using the COLOFIT logistic model rather than the Cox model results in slightly higher cost-effectiveness for COLOFIT compared with FIT driven by higher QALYs, but results are otherwise broadly similar. Adding additional colonoscopy harm means that QALYs are gained if risk stratification is used compared with referring everyone to USC, but COLOFIT still results in QALY loss compared with FIT, though cost-effectiveness results are similar.

**Table 2:** Comparison of incremental net monetary benefit per patient (based on a threshold of £20 000 per QALY) for base case and scenario analyses comparing FIT and COLOFIT thresholds against referring all urgently

Scenario	0.64% risk threshold		3% risk threshold	
	FIT 10	COLOFIT 0.64%	FIT 40	COLOFIT 3%
Base case	£464	£469	£517	£519
(1a) Include adenomas in the model	−£405	−£420	−£478	−£492
(1b) Include adenomas and IBD in the model	−£1016	−£1267	−£1506	−£1637
(2) Population based on Nottingham derivation cohort	£461*	£457*	£515*	£512*
(3) Use logistic COLOFIT algorithm	£463	£474	£517	£520
(4) Addition of colonoscopy harm	£491	£497	£548	£550
(5a) Doubled diagnostic delay	£459	£464	£509	£509
(5b) Faster stage transitions for delayed diagnoses	£441	£444	£472	£469
(5c) Combination of 5a and 5b	£432	£435	£457	£451
(6a) Discount rates increased to 5%	£465	£471	£519	£521
(6b) Discount rates reduced to 1.5%	£462	£467	£513	£514
(7) COLOFIT costs increased	£464	£462	£517	£511

Note that base case results vary slightly from those presented in table 1 as these are deterministic analyses.

* For this population, the FIT:COLOFIT comparison does not result in equal numbers of CRC diagnosis; if these are equalised, COLOFIT is more cost-effective than FIT at both thresholds.

COLOFIT, Algorithm developed for the COLOFIT project; CRC, colorectal cancer; FIT, fecal immunochemical test; IBD, inflammatory bowel disease; QALY, quality-adjusted life year.

Increasing the CRC stage shift caused by diagnostic delay increases QALY loss at all thresholds, reducing the optimal threshold and altering the balance between FIT and COLOFIT. If diagnostic delay is doubled and stage transitions are increased, the optimal threshold reduces to around the 2% risk level (online supplemental figure S2a, b). COLOFIT is still more cost-effective than FIT below this threshold, but at/above this threshold, FIT is more cost-effective than COLOFIT as the QALY loss in the young overwhelms the cost-savings caused by reduced USC referrals in the young. Altering discount rates has little impact on cost-effectiveness, as results are driven by USC referral costs, which are incurred in the first modelled year and are undiscounted. Increasing COLOFIT costs from additional blood tests has little impact on optimal threshold, but eliminates cost-savings of COLOFIT compared with FIT, resulting in little or no cost-effectiveness benefit.

## Discussion

Health economic analysis indicates that COLOFIT is likely to be cost-effective compared with using FIT alone at equivalent risk thresholds. The modelling also suggests that if applied, higher thresholds (for either FIT or COLOFIT) could potentially be more cost-effective than the current recommended threshold of 10 mg Hb/g, although there is greater uncertainty around COLOFIT cost-effectiveness compared with FIT at higher thresholds. In both cases, cost-effectiveness arises by saving costs through a reduction in the number of expensive USC referrals required to detect similar numbers of cancers; however, QALYs, LYs and lives are lost. The choice to use COLOFIT or higher risk thresholds would therefore technically represent a disinvestment decision. Disinvestment recommendations are still relatively uncommon in health technology assessment and can be controversial.^34^ However, in this particular case, the limitations on colonoscopy capacity mean that current levels of referral are unfeasibly high^5^ and are limiting expansion of other beneficial services such as CRC screening. Excessive referral numbers are resulting in additional diagnostic delay due to capacity constraints and therefore likely to be causing further QALY loss not captured in this modelling. COLOFIT and/or higher thresholds could therefore be used to reduce symptomatic referrals to a more sustainable level, retaining the same level of investment in colonoscopy services but potentially freeing up spare capacity for screening where CRC yield is higher (due to higher FIT thresholds) and at earlier stage of diagnosis.^35^

The situation is complicated further by our results showing that reduction in USC referrals and colonoscopies occurs predominately in younger patients. This is likely to be driven by the age variables in the COLOFIT algorithm. While this makes sense clinically given that CRC is much more prevalent in older people, it means that at any given risk threshold, expedited CRC diagnoses are reprioritised from the under 50s, who have greater remaining lifetime to benefit, to the over 70s, who have less remaining lifetime and lower health-related quality of life. This disadvantages the small number of young patients who transpire to have cancer and drives the observed net QALY loss. While the QALY loss is small, this could be underestimated given there is evidence that young people already suffer greater delays in diagnosis than do older patients.^36^ Furthermore, CRC in the young is increasing in incidence^37^ and considerable efforts are currently being made to try and improve diagnosis in this age group. We do not have data currently to improve the accuracy of clinical prediction in younger patients. The use of FIT tests in people aged under 50 is increasing and significant work is going on more widely aimed at risk stratifying possible CRC, though it is likely to be some years before a specific algorithm for younger patients can be generated and validated due to the small number of cancers identified in these groups. Such an algorithm may potentially allow more precise targeting of USC/colonoscopy resources, balancing the desire to undertake fewer investigations in low-risk younger people but potentially allowing the small number of cancers that exist to be diagnosed rapidly. If the COLOFIT model were to be adopted, therefore it is important that there is a robust plan to ‘safety-net’ all, but in particular those younger people not offered definitive investigation.

Given that much of the model data comes from Nottingham where the COLOFIT algorithm was developed and internally validated,^15^ it is worth considering whether COLOFIT could perform differently in other populations, which may have different underlying CRC prevalence, risk factor distribution or diagnostic pathways, and how this might impact the health economic outcomes. Our findings indicate that cost-effectiveness is strongly driven by the ability of the algorithm to prevent excess urgent referrals. This means that we can conclude as a general principle that for a given population, and at a particular CRC risk threshold, then COLOFIT is likely to be the most cost-effective option, providing it is able to significantly reduce referrals compared with FIT. Is this likely to be the case nationally? Previous development of an algorithm combining parameters for FIT and blood results did not show significant improvement on using FIT alone;^38^ however, there were significant problems in that study that were avoided during COLOFIT development and were more related to unconventional sample collection rather than any regional differences in population structure or clinical pathways (extensively discussed in Crooks *et al*^15^). Furthermore, COLOFIT performance has now been externally validated in two alternative regions of the UK (East Lancashire and Oxford), which has confirmed similar CRC prevalence across different populations and similar ability of COLOFIT to prevent unnecessary referrals in different local NHS systems.^15 16^ It is also worth considering whether the implementation of COLOFIT could prove more complex than modelled and introduce additional resource requirements or delays compared with FIT due to the need for a blood test. The cost scenario analysis presented here does suggest that costs saved from reduced colonoscopy must balance any additional costs incurred as a result of implementing COLOFIT for it to be cost-effective. Implementation analysis or piloting of COLOFIT is essential to enable such questions to be answered prior to any national roll-out.

Several important model uncertainties and limitations have been highlighted in the structural sensitivity analysis, which are important when making policy decisions. First, other serious bowel conditions such as IBD may present with similar symptoms and are detectable through FIT to a certain extent. These are worsened by diagnostic delay and may currently be diagnosed serendipitously but rapidly through the cancer referral process.^18^,^20^ Changes to the referral pathway are therefore likely to affect these non-cancer conditions. However, assessing the impact of COLOFIT on the diagnosis of non-cancer bowel conditions was out of the scope of the COLOFIT project, so while COLOFIT is likely to have some sensitivity for such diseases due to its large FIT component,^15^ it is unclear what the relative sensitivity and specificity of COLOFIT versus FIT might be. Our scenario analyses including adenomas and IBD in the model are therefore based on limited and uncertain data but suggest that COLOFIT could be less cost-effective than FIT if these conditions are taken into account. These results are likely to be driven by age variables in the COLOFIT algorithm promoting the reorientation of referrals to older people, as IBD is more prevalent in the young than cancer,^20^ and while adenomas are more prevalent in older people, their early detection may particularly benefit younger people by preventing cancer in later life. Further research into the likely impact of COLOFIT on the diagnosis of other serious bowel diseases, and the development of alternative referral pathways if the impact is likely to be significant, is therefore essential to ensure that people with these conditions are not disadvantaged.

A second important limitation is the ability of the model to estimate accurately the harms of delayed diagnosis. Studies looking at the impact of diagnostic delay have struggled with confounding given that people with higher risk of more advanced disease tend to be referred more urgently. Data from one study suggest that for people with high-risk symptoms, mortality could be 30–50% higher if diagnosis is delayed by more than 3 months, although this association was not seen for patients presenting with other symptoms,^4^ and has not been observed more generally across symptomatic patients in other studies.^39 40^ Rather than using this uncertain data, we took a natural history modelling approach whereby harms of delayed diagnosis were driven by stage shift based on transition probabilities calibrated as part of previous modelling work.^27^,^29^ However, this approach has its own limitations as it ignores disease progression within a stage, relies on estimates of diagnostic delay that omit recent increases in capacity constraints, and uses stage transition probabilities that were calibrated within a general population and therefore may not accurately represent how rapidly cancer develops in a symptomatic population. Our scenario analysis shows that increasing the impact of delay changes the balance of costs to QALYs, thereby reducing both the cost-effectiveness of COLOFIT compared with FIT and the optimal risk threshold. Assessing the impact on cancer outcomes would therefore be essential if COLOFIT and/or lower risk thresholds are implemented.

## Conclusion

Health economic modelling suggests that COLOFIT is likely to be more cost-effective than FIT at diagnosing a similar number of cancers and could help alleviate pressure on secondary care diagnostic services through significant reduction in the number of referrals required. However, the reorientation of expedited CRC diagnoses from people aged under 50 to those aged over 70, and resulting subsequent QALY loss, suggests that strategies to improve diagnosis in the under 50s are necessary to mitigate potential harm, particularly in the context of increasing incidence in this age group. Further research would enable uncertainties to be resolved around how COLOFIT might impact cancer survival and diagnosis of other serious bowel diseases in populations throughout England. Decisions around any future adoption of COLOFIT should consider cost-effectiveness alongside other priorities including diagnostic capacity, implementation practicalities and potential benefits and harms to different patient populations.

## Supplementary material

10.1136/bmjph-2024-002089 online supplemental file 1

10.1136/bmjph-2024-002089 online supplemental file 2

10.1136/bmjph-2024-002089 online supplemental file 3

## Data Availability

Data are available upon reasonable request.

## References

[1] (2022). Bowel cancer statistics: cancer research UK. https://www.cancerresearchuk.org/health-professional/cancer-statistics/statistics-by-cancer-type/bowel-cancer/.

[2] (2022). Early diagnosis data hub: cancer research UK cancer intelligence team. https://crukcancerintelligence.shinyapps.io/EarlyDiagnosis/.

[3] NICE (2023). NG12 suspected cancer: recognition and referral. https://www.nice.org.uk/guidance/ng12.

[4] Arhi CS, Burns EM, Bottle A (2020). Delays in referral from primary care worsen survival for patients with colorectal cancer: a retrospective cohort study. Br J Gen Pract.

[5] Ravindran S, Thomas-Gibson S, Bano M (2022). National census of UK endoscopy services 2021. Frontline Gastroenterol.

[6] NHS (2021). Overview: bowel cancer screening. https://www.nhs.uk/conditions/bowel-cancer-screening/.

[7] Gavin DR, Valori RM, Anderson JT (2013). The national colonoscopy audit: a nationwide assessment of the quality and safety of colonoscopy in the UK. Gut.

[8] Ismail MS, Aoko O, Sihag S (2020). Lower gastrointestinal symptoms and symptoms-based triaging systems are poor predictors of clinical significant disease on colonoscopy. BMJ Open Gastroenterol.

[9] Bailey SER, Abel GA, Atkins A (2021). Diagnostic performance of a faecal immunochemical test for patients with low-risk symptoms of colorectal cancer in primary care: an evaluation in the South West of England. Br J Cancer.

[10] Chapman C, Bunce J, Oliver S (2019). Service evaluation of faecal immunochemical testing and anaemia for risk stratification in the 2-week-wait pathway for colorectal cancer. BJS Open.

[11] D’Souza N, Georgiou Delisle T, Chen M (2021). Faecal immunochemical test is superior to symptoms in predicting pathology in patients with suspected colorectal cancer symptoms referred on a 2WW pathway: a diagnostic accuracy study. Gut.

[12] Monahan KJ, Davies MM, Abulafi M (2022). Faecal immunochemical testing (FIT) in patients with signs or symptoms of suspected colorectal cancer (CRC): a joint guideline from the Association of Coloproctology of Great Britain and Ireland (ACPGBI) and the British Society of Gastroenterology (BSG). Gut.

[13] NICE (2023). DG56 quantitative faecal immunochemical testing to guide colorectal cancer pathway referral in primary care. https://www.nice.org.uk/guidance/dg56.

[14] Hampton JS, Kenny RPW, Rees CJ (2023). The performance of FIT-based and other risk prediction models for colorectal neoplasia in symptomatic patients: a systematic review. EClinicalMedicine.

[15] Crooks CJ, West J, Jones J (2025). COLOFIT: Development and Internal-External Validation of Models Using Age, Sex, Faecal Immunochemical and Blood Tests to Optimise Diagnosis of Colorectal Cancer in Symptomatic Patients. Aliment Pharmacol Ther.

[16] Tamm A, Shine B, James T (2025). External validation of the colofit colorectal cancer risk prediction model in the oxford-fit dataset: the importance of population characteristics and clinically relevant evaluation metrics.

[17] (2024). R: a language and environment for statistical computing. https://www.R-project.org/.

[18] Nguyen VQ, Jiang D, Hoffman SN (2017). Impact of Diagnostic Delay and Associated Factors on Clinical Outcomes in a U.S. Inflammatory Bowel Disease Cohort. *Inflamm Bowel Dis*.

[19] Walker GJ, Lin S, Chanchlani N (2020). Quality improvement project identifies factors associated with delay in IBD diagnosis. Aliment Pharmacol Ther.

[20] D’Souza N, Monahan K, Benton SC (2021). Finding the needle in the haystack: the diagnostic accuracy of the faecal immunochemical test for colorectal cancer in younger symptomatic patients. Colorectal Dis.

[21] Wong MCS, Huang J, Huang JLW (2020). Global Prevalence of Colorectal Neoplasia: A Systematic Review and Meta-Analysis. Clin Gastroenterol Hepatol.

[22] NHS England (2022). 2021/22 national cost collection data. https://www.england.nhs.uk/costing-in-the-nhs/national-cost-collection/.

[23] Bellini D, Rengo M, De Cecco CN (2014). Perforation rate in CT colonography: a systematic review of the literature and meta-analysis. Eur Radiol.

[24] Gatto NM, Frucht H, Sundararajan V (2003). Risk of perforation after colonoscopy and sigmoidoscopy: a population-based study. J Natl Cancer Inst.

[25] Rutter MD, Nickerson C, Rees CJ (2014). Risk factors for adverse events related to polypectomy in the English Bowel Cancer Screening Programme. Endoscopy.

[26] Lyratzopoulos G, Abel GA, McPhail S (2013). Measures of promptness of cancer diagnosis in primary care: secondary analysis of national audit data on patients with 18 common and rarer cancers. Br J Cancer.

[27] Mandrik O, Thomas C, Strong M (2021). Calibration and validation of the microsimulation model in cancer of the bowel (MiMiC-Bowel), an individual patient simulation model for investigation of the cost-effectiveness of personalised screening.

[28] Thomas C, Mandrik O, Whyte S (2020). Development of the microsimulation model in cancer of the bowel (MiMiC-Bowel), an individual patient simulation model for investigation of the cost effectiveness of personalised screening and surveillance strategies.

[29] Thomas C, Mandrik O, Whyte S (2022). Modelling cost-effective strategies for minimising socioeconomic inequalities in colorectal cancer screening outcomes in England. Prev Med.

[30] (2019). Cancer survival in England - adults diagnosed 2013-2017.

[31] NHS England (2020). Routes to diagnosis: colorectal cancer: incidence: national disease registration service. https://nhsd-ndrs.shinyapps.io/routes_to_diagnosis/.

[32] (2021). National life tables: England. https://www.ons.gov.uk/peoplepopulationandcommunity/birthsdeathsandmarriages/lifeexpectancies/datasets/nationallifetablesenglandreferencetables.

[33] National Institute for Health and Care Excellence (NICE) (2013). Guide to the methods of technology appraisal. https://www.nice.org.uk/process/pmg9/resources/guide-to-the-methods-of-technology-appraisal-2013-pdf-2007975843781.

[34] Kamaruzaman HF, Grieve E, Wu O (2022). Disinvestment in healthcare: a scoping review of systematic reviews. Int J Technol Assess Health Care.

[35] Beaton D, Sharp L, Lu L (2024). Diagnostic yield from symptomatic lower gastrointestinal endoscopy in the UK: A British Society of Gastroenterology analysis using data from the National Endoscopy Database. Aliment Pharmacol Ther.

[36] Rydbeck D, Asplund D, Bock D (2021). Younger age at onset of colorectal cancer is associated with increased patient’s delay. Eur J Cancer.

[37] Vuik FE, Nieuwenburg SA, Bardou M (2019). Increasing incidence of colorectal cancer in young adults in Europe over the last 25 years. Gut.

[38] Withrow DR, Shine B, Oke J (2022). Combining faecal immunochemical testing with blood test results for colorectal cancer risk stratification: a consecutive cohort of 16,604 patients presenting to primary care. BMC Med.

[39] Murchie P, Raja EA, Brewster DH (2014). Time from first presentation in primary care to treatment of symptomatic colorectal cancer: effect on disease stage and survival. Br J Cancer.

[40] Padilla-Ruiz M, Morales-Suárez-Varela M, Rivas-Ruiz F (2022). Influence of Diagnostic Delay on Survival Rates for Patients with Colorectal Cancer. Int J Environ Res Public Health.

