# Supplementary material for: Cost-effectiveness of FIT and a FIT-based model to optimise symptomatic diagnosis of colorectal cancer: health economic modelling for the COLOFIT project
Source: BMJ Public Health. 2025 Jun 24;3(1):e002089. doi: 10.1136/bmjph-2024-002089 (PMC12198831; doi:10.1136/bmjph-2024-002089)
Supplement: online supplemental file 1 [file bmjph-3-1-s001.docx]

**Cost-effectiveness of FIT and a FIT-based model to optimise symptomatic diagnosis of colorectal cancer: health economic modelling for the COLOFIT project**

**Supplementary Technical Methods**

Contents

[1. Conceptual Modelling 2](#_Toc177649906)

[2. Model Structure 2](#_Toc177649907)

[3. Model Population 3](#_Toc177649908)

[Baseline Population 3](#_Toc177649909)

[Assigning Health States and Health-Related Quality of Life 5](#_Toc177649911)

[4. Short-term Diagnostics Model 9](#_Toc177649915)

[Short-term Model Probabilities 10](#_Toc177649916)

[Short-term Model Costs & Utilities 14](#_Toc177649920)

[5. Long-term Markov Model 17](#_Toc177649923)

[Long-Term Model Transition Probabilities 17](#_Toc177649924)

[Long-term Model Costs and Utilities 20](#_Toc177649928)

[Integrating Short and Long-term Models 22](#_Toc177649931)

[6. Model Outcomes 23](#_Toc177649932)

[7. Model Validation 24](#_Toc177649933)

[8. All Model Parameters & Distributions 26](#_Toc177649934)

[9. References 33](#_Toc177649935)

# Conceptual Modelling

Conceptual modelling was carried out prior to model development to define the research problem, develop an appropriate model structure, make decisions around appropriate sources and values for model parameters, and decide upon key model outcomes and analyses. The resulting document was circulated among members of the wider COLOFIT project team for input and discussion. This resulted in identification of additional data sources and prioritisation of model outcomes and analyses. Modelling plans also evolved in response to findings from other COLOFIT work packages, data availability and feasibility/practicality. As a consequence, the initial conceptual modelling document was regularly updated throughout model development and eventually became this technical methods document detailing what was actually done.

Patient and public involvement (PPI) was an important part of the COLOFIT project as a whole, but had a lesser role in the health economic modelling. PPI input was not sought at the conceptual modelling phase. However, model results were presented to the PPI panel prior to publication.

# Model Structure

The COLOFIT health economic model is an individual patient level simulation, with a short-term model which simulates the process and outcomes of initial diagnosis, and a long-term Markov style model with annual cycles which models the long-term impacts of disease (Figure 1).

Figure 1: Model structure indicating the key processes that occur in the short-term and long-term model.


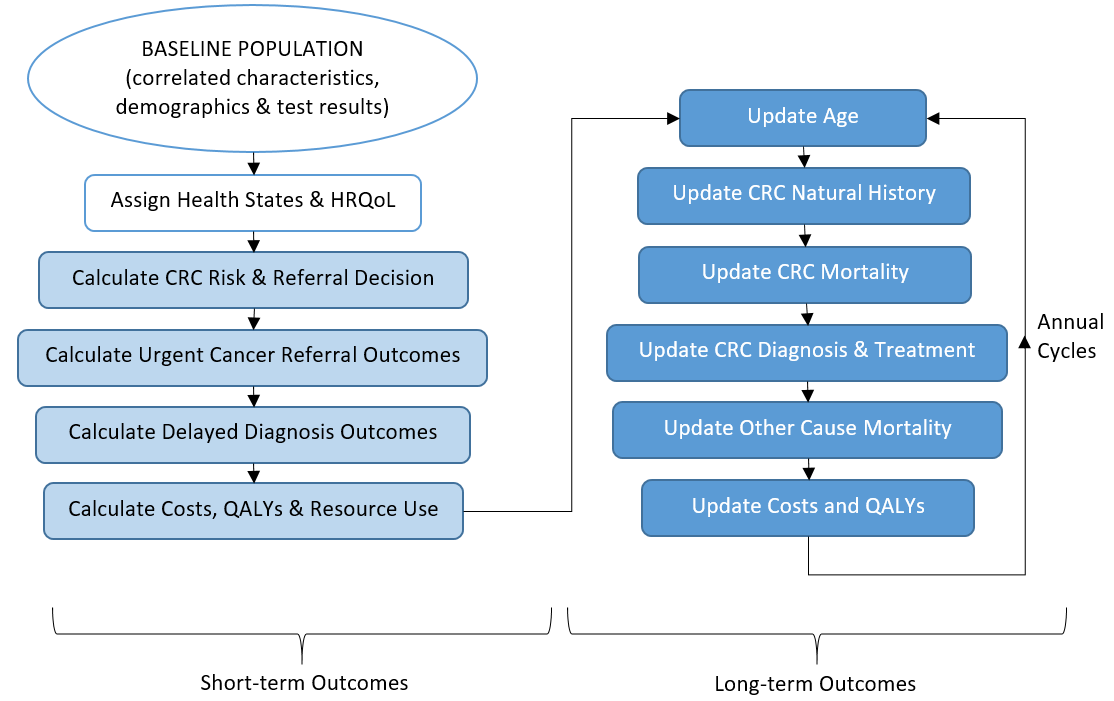


The model starts with a baseline population of individual patients, who have a set of correlated personal characteristics, which include demographic information such as age and sex, and test results such as FIT and blood tests. Underlying health states are then assigned to each individual based on their personal characteristics. This includes a set of mutually exclusive health states determining their colorectal cancer (CRC) natural history status, plus the health state of inflammatory bowel disease (IBD), which is assigned separately. Health-related quality of life is then assigned to each person dependant on their personal characteristics and underlying health state.

Once the model population is set up, the short-term model decides what happens to each individual within the year of diagnosis from the point of the index FIT sample being taken following GP consultation. This includes estimation of CRC risk, referral decision and the process and outcome of patients who undergo an urgent suspected cancer (USC) referral, or who have a delayed diagnosis of CRC, adenomas or IBD. Costs, quality-adjusted life years (QALYs) and resource use are then calculated for the process of diagnosis. Short-term results are then fed into the long-term model, which annually updates age and CRC natural history transitions including cancer development, progression, diagnosis, treatment and mortality, enabling the long-term cost and quality of life impacts of differential diagnostic criteria to be assessed. Long and short term outcomes are combined to obtain final cost-effectiveness results.

# Model Population

## Baseline Population

The target population is comprised of all adult patients attending their GP in England, with symptoms that give the GP reason to consider CRC as a possible diagnosis. Defining precisely who should be included in this group is not straightforward as it is likely to vary between local areas and GP practices; however it should include both the high-risk and low-risk groups specified in the previous NICE guidance DG30 and NG151 [1, 2], and will also include anyone currently being sent for symptomatic FIT (which may include some additional lower risk people, but exclude some people with very high risk symptoms such as rectal mass). This is referred to as the pan-risk or pan-FIT population.

For model purposes, the baseline population characteristics were taken from the Nottingham dataset, with separate model populations constructed from the derivation dataset, which was used to build the COLOFIT algorithm, and the validation dataset, which was used for internal-external validation of the algorithm. The validation population was used to produce basecase model results, whilst the derivation population was used for sensitivity analysis. The derivation dataset consists of 34,231 people who were sent for symptomatic FIT by their GP in Nottingham between 2016 and 2021, and who had a valid FIT score and at least one year of follow-up; whilst the validation population consists of 16,735 equivalent people, with data collected between 2021 and 2022. Data from a service evaluation of the first year of symptomatic FIT usage has been published [3].

The Nottingham dataset should approximate the target population reasonably well; however, it does not include data on people who were advised to take a FIT but did not do it (some information on these individuals is available [4], but not included in this analysis). Note also that people who do not present to the GP with symptoms and instead are diagnosed through emergency presentation or only after death are not represented. These people do not impact on the decision problem, so excluding them from the model is appropriate; however, the composition of the population may change if symptomatic presentation or FIT uptake rates were to differ from those represented in the dataset. Included population characteristics from the Nottingham dataset comprised demographic information and the risk variables included in the COLOFIT algorithm. The following variables were included: age, sex, ethnicity, socioeconomic deprivation quintile, FIT score, platelet count, mean corpuscular volume (MCV) and haemoglobin count.

### Construction of synthetic baseline population

Given that the actual, patient level data were not available to the health economics team, and to enable the model to be free of data protection requirements, synthetic populations were constructed based on aggregate information from the derivation and validation datasets (extracted by the COLOFIT algorithm development team in Nottingham). This used mean values/distributions and correlation information (in the format of variance-covariance matrices) to sample the population, assuming that all variables followed a multivariate normal distribution. However, many of the included variables were highly skewed. In order to adjust for this, certain variables were transformed ahead of synthetic population sampling so as to better approximate a normal distribution. This included age, MCV and haemoglobin count, all incorporated as squared terms; FIT, incorporated as a log term, and platelet count, incorporated as a square-rooted term.

Multivariate sampling of transformed variables alone was insufficient to construct an adequate population due to some people being assigned implausible values. To prevent this from happening, and improve the fit of the synthetic population to the data, sampled values were converted into quantiles and then constrained using a series of cut-offs to fit quantile data from the Nottingham population, before being transformed back into values. This was particularly important for FIT values, given its particularly high importance in risk prediction. Given an observed interaction between FIT and age, FIT percentiles were extracted from the Nottingham datasets for three different age groups in the population (age <50, age 50-70 and age 70+), and then modelled quantile data was fitted precisely within these cut-offs. Other variables were categorical (e.g. sex, ethnicity & IMD), and these were assigned from the multivariate normal distribution based on a series of cut-offs corresponding to the proportion in each category.

One million synthetic individuals from each population (derivation and validation) were pre-generated for use in the model of which a subset were randomly selected for each model run. A summary of the personal characteristics for the synthetic populations can be seen in Table 1.

Table 1: Summary statistics for the synthetic baseline populations (n = 1 million)

| **Characteristic** | **Derivation Population** | **Validation Population** |
| --- | --- | --- |
|  | **Mean (Standard Deviation)** | |
| Age (years) | 66.23 (15.07) | 62.74 (15.66) |
| MCV (femtolitres) | 91.94 (7.70) | 92.71 (5.62) |
| Haemoglobin count (g/L) | 123.6 (21.6) | 129.8 (18.4) |
| Platelet count (x10^9^/L) | 283.5 (93.6) | 269.8 (66.9) |
|  | **Percentage** | |
| Male Sex | 45.3% | 44.7% |
| Ethnic Group White | 71.3% | 69.3% |
| Ethnic Group Asian | 3.8% | 3.9% |
| Ethnic Group Black | 2.8% | 2.9% |
| Ethnic Group Other | 2.6% | 2.8% |
| Ethnic Group Not Reported | 19.6% | 21.0% |
| IMD1 (least deprived) | 15.3% | 15.4% |
| IMD2 | 15.8% | 16.8% |
| IMD3 | 37.2% | 35.5% |
| IMD4 | 14.0% | 14.1% |
| IMD5 (most deprived) | 17.7% | 18.2% |
| FIT > 4 µg Hb/g | 33.3% | 31.6% |
| FIT ≥ 10 µg Hb/g | 22.7% | 23.5% |
| FIT ≥ 40 µg Hb/g | 10.4% | 10.1% |
| MCV mean corpuscular volume; IMD index of multiple deprivation; FIT faecal immunochemical test; Hb haemoglobin. | | |

## Assigning Health States and Health-Related Quality of Life

Every individual in the baseline population is assigned health states at model start, which depend upon their personal characteristics and the estimated prevalence of disease in the population. The model has two sets of health states; a set of mutually exclusive health states relating to the CRC pathway, and a separate health state relating to whether an individual has IBD or not (Figure 2).

### Disease Prevalence

There is limited data about the underlying prevalence of disease in a symptomatic population sent a FIT, as most individuals will not undergo further investigation and hence adenomas and even more serious disease may go undetected for quite some time. The Nottingham dataset provided information that could be used to approximate the underlying prevalence of CRC from one year follow-up for cancer outcomes. Overall one-year CRC prevalence was 1.5% in the derivation population and 1.1% in the validation population, although these varied by age group (see Table 2).

Figure 2: Diagram indicating the relationship between population characteristics (pink boxes) and disease health states (green and blue boxes).


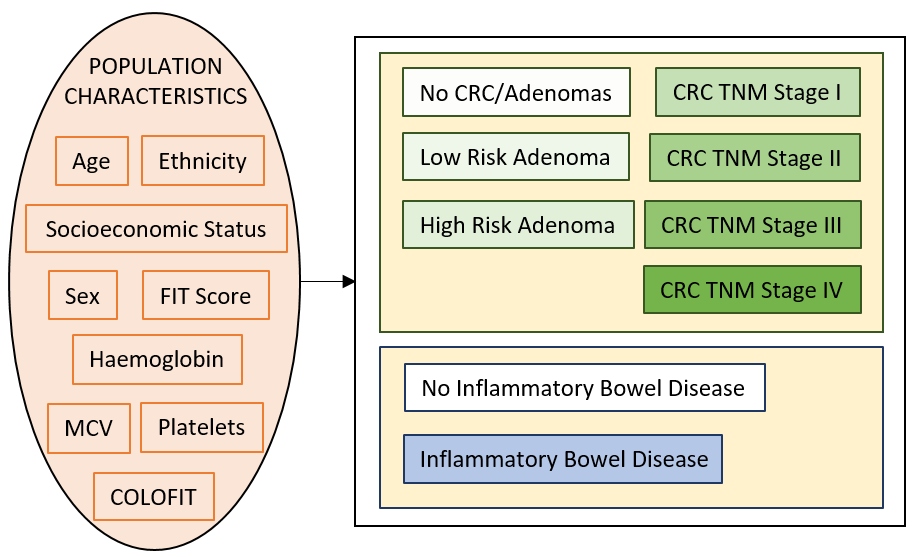


These values were not used directly as inputs in the model; instead, prevalence of CRC at baseline was directly determined through application of the risks given through the COLOFIT algorithm to the individual patients in the synthetic population (described below). Note that the COLOFIT algorithm is derived using one-year follow-up data and therefore using this algorithm to determine model baseline prevalence of CRC relied on assumptions that: 1) all CRC cases present at baseline were diagnosed within a year; 2) all cancers diagnosed within a year were present at baseline. Whilst these assumptions are unlikely to be true, they have opposite impacts on prevalence therefore may cancel out to a certain extent.

Nottingham data from the derivation population was also used to inform stage distribution at diagnosis, based on 533 CRC cases. Whilst this was data was available by age, small numbers meant that no significant differences by age could be observed, so it was assumed that stage distribution did not vary by age or by any of the other characteristics in the modelled population. For a certain proportion of the CRC cases, stage data was missing. There is evidence suggesting that survival in people with missing stage data is very poor in the short-term, but from year one onwards after diagnosis is slightly better than in people with stage IV cancer [5]. It was therefore assumed that cancers with missing stage data would be distributed between stage III and IV in the same ratio as the recorded stage III/IV cancers.

To inform total prevalence of both high and low risk adenomas in the population sent for FIT, a NICE FIT steering group authored study of 9,822 patients with suspected CRC sent for further investigation via USC, between 2017 and 2019 in England was used [6]. This population differs from the modelled population as individuals sent for USC investigation have higher CRC risk than those sent for FIT, and indeed CRC prevalence was higher than in the Nottingham population at 3.3%. However, adenoma prevalence was similar in a study of the general population [7], suggesting that adenoma prevalence may not correlate particularly well with symptoms (Table 2).

Table 2: Prevalence data used for assigning and validating health states in the model baseline population

| **Health State** | **Mean Prevalence** | **Lower 95% CI** | **Upper 95% CI** | **Source** |
| --- | --- | --- | --- | --- |
| CRC Age < 50 (1 year) | 0.004 | 0.003 | 0.006 | Nottingham Derivation Population |
| CRC Age 50-70 (1 year) | 0.010 | 0.008 | 0.012 |  |
| CRC Age 70+ (1 year) | 0.024 | 0.022 | 0.027 |  |
| CRC all ages (1 year) | 0.015 | 0.014 | 0.016 |  |
| CRC all ages (1 year) | 0.011 | 0.009 | 0.012 | Nottingham Validation Population |
| CRC Stage I (of all CRC) | 0.213 | 0.179 | 0.249 | Nottingham Derivation Population |
| CRC Stage II (of all CRC) | 0.157 | 0.127 | 0.189 |  |
| CRC Stage III (of all CRC) | 0.313 | 0.274 | 0.353 |  |
| CRC Stage IV (of all CRC) | 0.187 | 0.155 | 0.222 |  |
| CRC Stage Missing (of all CRC) | 0.130 | 0.103 | 0.160 |  |
| High Risk Adenoma | 0.043 | 0.039 | 0.047 | D’Souza 2021a [6] |
| Low Risk Adenoma | 0.236 | 0.228 | 0.245 | D’Souza 2021a [6] |
| Adenoma >5mm in FIT≥10 | 0.115 | 0.108 | 0.122 | Nottingham Derivation Population |
| Adenoma >5mm in FIT<10 | 0.015 | 0.014 | 0.017 |  |
| IBD Age <50 | 0.079 | 0.064 | 0.095 | D’Souza 2021b [8] |
| IBD Age >50 | 0.039 | 0.035 | 0.043 | D’Souza 2021b [8] |

Nottingham data was used to help inform how prevalence of high risk adenomas varied by FIT status. Information was available about the one-year prevalence of adenomas bigger than 5mm, separately in the populations with FIT≥10, for which it was 11.5%, and with FIT<10, for which it was 1.5% (Table 2). This suggests that FIT (and therefore potentially COLOFIT) has some sensitivity for large adenomas, although the prevalence is expected to be underestimated in the FIT<10 population as it is not expected that all adenomas will be detected within a year. Note that the definition of high risk adenoma uses several criteria and is not equivalent to adenomas >5mm; however for the purposes of the model it was assumed to be comparable. Combining Nottingham data about prevalence in the FIT≥10 population, with the total HR adenoma prevalence estimates from the NICE USC study described above [6], enabled prevalence in the FIT<10 population to be recalculated as 2.1%. In the absence of any Nottingham data around low risk adenomas, it was assumed that prevalence would not vary by FIT status. Note that colonoscopy has imperfect sensitivity for detecting adenomas (see below), so the input prevalence values were adjusted in the model to reflect this.

The NICE USC study also indicated that overall IBD prevalence in people with suspected CRC is 4.3% [6]. However, it was important to capture differences in prevalence by age in the modelled baseline population to enable harms of delayed diagnosis to be captured accurately. A study of the same population looking at age differences, was used to inform prevalence of IBD in people aged under and over 50 [8] (Table 2).

### Assigning Baseline Health States

Modelled individuals were assigned to mutually exclusive CRC natural history health states, and then IBD status was assigned afterwards. The COLOFIT cox survival model including blood results was used to determine CRC risk for each individual in the baseline population, as this was the most accurate COLOFIT model developed by the Nottingham team, although the logistic model was also tested in sensitivity analysis. CRC status was assigned randomly to the population based on their underlying risk. Internal validation was carried out to check that this resulted in the expected population prevalence of CRC. Stage was then assigned randomly to those with CRC.

The COLOFIT algorithm is likely to have some predictive power for HR adenomas, although this has not been assessed. CRC risk as predicted through the COLOFIT algorithm was used to determine which individuals would be assigned the HR adenoma health state, of those not already assigned to CRC. This was done by scaling risk in individuals with FIT ≥10 to adenoma >5mm prevalence data, and then scaling risk in individuals with FIT<10 to obtain the expected overall HR adenoma prevalence. It was assumed predictive power for LR adenomas was no better than random so LR adenoma health state was assigned randomly to people without either CRC or HR adenoma to reach the expected prevalence. All other people were assumed to be in the normal epithelium health state with respect to the CRC pathway.

It was assumed that nobody had more than one bowel disease at baseline, so the IBD health state was applied only to people with no CRC or adenomas. FIT has some sensitivity for IBD, and the paper used to inform IBD prevalence also reported positive predictive values for IBD at three different FIT thresholds in people aged >50 and <50 [8]. This data was used to assign IBD status randomly to individuals in groups based on their age and FIT score. Note that correlation with FIT implies that the COLOFIT algorithm is likely to have some sensitivity to IBD, but this has not been formally tested.

### Assigning Baseline Health-Related Quality of Life Scores

In order to calculate QALYs as a model outcome, all individuals required a health-related quality of life (HRQoL) score at baseline. This score has a maximum value of 1 (perfect quality of life), whilst 0 represents no quality of life (or death). HRQoL varies by age and underlying condition. Given that individuals are not diagnosed at the point that the model starts, it was assumed that the utility decrement caused by symptoms would be roughly similar across all health states. A study of health state utility values from the general population found that mean HRQoL value for individuals with bowel symptoms and an average age of 53 was 0.7 (SD = 0.0168), compared to 0.83 for individuals without bowel symptoms [9]. The same study indicates that there is a roughly linear relationship between age and HRQoL, with every increasing year of age being associated with a utility decrement of 0.00444 (SD = 0.0018). Modelled individuals were each assigned a baseline HRQoL based on random sampling of the bowel symptoms distribution plus/minus a randomly sampled age decrement corresponding to their age at baseline. Both distributions were assumed to be normal, but resulting individual baseline HRQoL values were truncated at 0 and 1.

# Short-term Diagnostics Model

The short-term diagnostics model represents a year of symptomatic presentation for CRC. It is a simple simulation that steps through a series of different diagnostic processes in turn and uses a series of random draws to determine who undergoes which processes, based on individual level probabilities. A schematic of the process is shown in Figure 1A of the main manuscript, and briefly described below.

Everyone in the symptomatic population was assumed to visit the GP and undergo FIT. Risk stratification took place either based on FIT score alone or a COLOFIT algorithm, and as a result individuals were either sent down the USC pathway or would not undergo a USC referral. Individuals who were not immediately referred for USC may have had no further contact with the health services for their symptoms, may have returned to the GP and be referred at a later date (through unspecified referral pathways that may be USC or non-urgent), or may have presented as an emergency (CRC only). Referral to secondary care through any of these routes involved the opportunity to undergo colonoscopy or CTC, and depending upon underlying health state, be diagnosed with CRC, IBD or adenomas. Note that treatment for CRC and IBD was not incorporated in the short-term model, but polypectomy for adenomas was included. It was assumed in the model that anyone with CRC or IBD at baseline would be diagnosed through one of these routes in the short-term model, whereas individuals with adenomas may or may not remain undiagnosed. Most of these diagnostic processes use NHS resources and incur costs, and some processes also incur quality of life decrements.

Whilst the short-term model incorporates some events that occur in subsequent years (due to delayed diagnosis), it would be expected that in any given year, diagnoses would occur that have been delayed following initial symptomatic presentation in previous years. It was therefore possible to estimate the annual resource requirements, costs and utility decrements incurred for each diagnostic scenario once it had reached steady state in the population. Discounting was therefore not applied in the short-term model to enable these annual outcomes to be represented.

## Short-term Model Probabilities

### GP Attendance & Risk Stratification

All modelled symptomatic patients were assumed to attend their GP initially and undergo FIT. Whilst in practice some individuals do not uptake FIT [3, 4, 10], the Nottingham dataset does not represent these people, nor would their results differ by model arm, so they were ignored for modelling purposes. Some repeat FITs were required due to inadequate first samples, however, these were not modelled explicitly and instead were included only in the resource use and cost calculations (see Short-term Model Costs). It was assumed that assigned individual FIT score was identical to the FIT result upon testing. In the current care arm, risk stratification depended upon FIT score alone, whereas in the COLOFIT arms, the COLOFIT algorithm including blood results was run again to determine risk (note that unlike for assigning health states, the algorithm variables were used as constants to reflect likely use in practice). It was assumed that all individuals who required blood tests took them up, were risk stratified and were referred via USC or not as appropriate. Validation was carried out to compare performance of FIT and COLOFIT in the health economic model with the test characteristics reported by the Nottingham team [11] (see Model Validation section).

### Urgent Suspected Cancer Pathway

All individuals referred to the USC pathway are assumed to be offered either colonoscopy or computed tomography colonography (CTC). Some patients may be offered other diagnostic modalities in practice (e.g. flexible sigmoidoscopy), but this is a small number of patients and so was not considered in the model. There is little good data to inform the probability of being offered CTC rather than colonoscopy and it is likely to vary highly by local area and patient age and fitness. An estimate of 11.7% was derived through expert elicitation for the previous NICE diagnostic assessment work on symptomatic FIT for triage of suspected CRC patients [12], and this was applied randomly to determine who would be offered CTC, with all other patients offered colonoscopy.

Colonoscopy and CTC have imperfect uptake and sensitivity, and this was incorporated in the modelling of the USC pathway, using English data sources based on USC or symptomatic detection where possible, found through rapid review (Table 3). Nottingham data was available to inform some of these parameters; however, it wasn’t directly used as patient numbers tended to be smaller than alternative sources which would have increased model uncertainty. Instead, Nottingham values were checked against these sources to ensure they were similar. Random draw was used to determine who took up follow-up investigations and whether or not an individual was diagnosed. Diagnosis was assumed to happen fairly shortly after the index GP appointment and therefore disease was assumed not to progress from the baseline health state. CTC frequently requires endoscopic follow-up. This includes all individuals who are thought to have CRC/adenomas after CTC (including false positives), and a number of inadequate CTC procedures. It was assumed in the model that all individuals with CRC/adenomas/IBD found through CTC would require an additional colonoscopy, together with a proportion of people with normal epithelium who represented the false positives and inadequate results. Inadequate/incomplete colonoscopy was also included in the model, which was assumed to require a repeat colonoscopy. Unlike the FIT inadequates, these were modelled explicitly to enable the total colonoscopy use to be estimated.

Table 3: Participation rates, test characteristics and harms for follow-up with colonoscopy/CTC

| **Parameter** | **Mean** | **Lower 95% CI** | **Upper 95% CI** | **Reference & Notes** |
| --- | --- | --- | --- | --- |
| Colonoscopy Uptake | 0.981 | 0.978 | 0.983 | D’Souza 2021a [6] based on % who cancelled follow-up |
| Colonoscopy Sensitivity for LR adenomas | 0.765 | 0.733 | 0.796 | Van Rijn 2006 [13]. Due to lack of data, based on mixture of screening & symptomatic colonoscopy in Netherlands. |
| Colonoscopy Sensitivity for HR adenomas | 0.925 | 0.894 | 0.952 | Martin Lopez 2014 [14]. Due to lack of data, based on screening colonoscopy. |
| Colonoscopy Sensitivity for CRC | 0.965 | 0.917 | 0.993 | Than 2015 [15] based on PCCRC rate. |
| Colonoscopy Sensitivity for IBD | 1 | 1 | 1 | Assumption based on gold standard for other tests. |
| Colonoscopy False Positive Rate | 0 | 0 | 0 | Assumption based on nature of test |
| Colonoscopy Incompletes/ Inadequates | 0.101 | 0.095 | 0.106 | D’Souza 2021a [6] based on % with incomplete colonoscopy |
| CTC Uptake | 0.967 | 0.959 | 0.975 | Stephenson 2018 [16] based on appointments offered & attended |
| CTC Sensitivity for LR adenomas | 0.627 | 0.381 | 1.018 | Atkin 2013 [17]. Assumption based on detection rates relative to colonoscopy |
| CTC Sensitivity for HR adenomas | 0.759 | 0.465 | 1.218 | Atkin 2013 [17]. Assumption based on detection rates relative to colonoscopy |
| CTC Sensitivity for CRC | 0.945 | 0.577 | 1.509 | Atkin 2013 [17]. Assumption based on detection rates relative to colonoscopy |
| CTC Sensitivity for IBD | 0.84 | 0.791 | 0.891 | Horsthuis 2008 [18]. Meta-analysis |
| CTC False Positive Rate/Inadequates | 0.197 | 0.164 | 0.232 | Atkin 2013 [17]. Based on % additional procedures required in those found not to have CRC/HR adenomas. |
| Therapeutic colonoscopy perforation rate | 0.00091 | 0.00061 | 0.00128 | Rutter 2014 [19]. Based on screening patients. |
| Diagnostic colonoscopy perforation rate | 0.00031 | 0.00014 | 0.00054 | Rutter 2014 [19]. Based on screening patients. |
| CTC perforation rate | 0.0008 | 0.000 | 0.0030 | Bellini 2014 [20]. Based on symptomatic patients. |
| Hospitalisation rate due to bleeding: colonoscopy | 0.0005 | 0.0003 | 0.0009 | Gavin 2013 [21]. Based on all patients. |
| Death rate: colonoscopy | 0.000031 | 0.00001 | 0.00008 | Tomaszweski 2021 [22]. Due to lack of data, based on screening patients from Canada. |
| Death rate: CTC | 0 | 0 | 0 | Bellini 2014 [20]. Based on all patients. |
| CTC computed tomography colonography; CRC colorectal cancer; IBD inflammatory bowel disease; LR low risk; HR high risk; CI confidence interval; PCCRC Post colonoscopy colorectal cancer; no. number. | | | | |

People diagnosed with CRC or adenomas were assumed to undergo therapeutic colonoscopy which would include biopsy or adenoma removal, whereas other colonoscopy was assumed to be diagnostic. Both CTC and colonoscopy have a small risk of adverse events such as bleeding, perforation and even death, with therapeutic colonoscopy having higher risks than diagnostic colonoscopy or CTC (Table 3). These were incorporated in the model, with random draw used to determine which individuals would suffer from adverse events.

### Delayed Diagnosis Pathway

Current recommendations suggest that patients who are not referred immediately via the USC pathway should undergo safety netting to ensure that patients with cancer are not missed [23]. However, the process of safety netting is poorly defined, likely to vary considerably by local area, and be highly heterogeneous, based on a combination of people not referred at all, those referred less urgently and those who may return to the GP on a later date and be referred urgently or less urgently at that point. If the safety netting fails, then people may present as an emergency. Due to the complexity and uncertainty around these pathways, a single ‘delayed diagnosis’ pathway was modelled for everyone not diagnosed through the USC pathway outlined above.

It was assumed that all individuals with CRC or IBD at baseline would be diagnosed within the short-term model; however, this would only occur after a delay, which would allow disease to progress in the intervening period. This was done in a slightly different way for each disease, which reflected the modelled natural history. Data was not available from Nottingham to inform this.

For CRC, each person who had not been diagnosed at USC was assigned an individual time to diagnosis based on random sampling from a distribution obtained from a UK primary care retrospective cohort study [24]. This suggested that of patients not diagnosed within two weeks, half would be diagnosed within 3 months, and half longer than three months. Three months was considered to be the mean value in a lognormal distribution, with the 99.99% quantiles assumed to be between two weeks and two years. During this time, transition to a more severe cancer stage could occur, based on natural history transition probabilities (see Long-term Markov Model section). This enabled the non-linear impact of potentially long delays occurring in a small proportion of individuals to be incorporated in the modelling.

IBD is a relapsing-remitting disease, with patients moving between mild, moderate and severe disease states characterised by the intensity of symptoms and being at risk for a range of complications including perforated bowel, severe inflammation or fistula [25, 26]. The complex relapsing-remitting nature of the disease was not modelled in detail and instead a simple probability of complicated disease versus non-complicated disease was assigned to diagnosed individuals, with delayed diagnosis of IBD assuming to contribute to an increased probability of complications irrespective of how long the delay actually was.

The probability of being diagnosed with IBD in different time periods after symptom onset, and the proportion of symptomatic IBD patients with complications were obtained from a UK study of 304 newly diagnosed IBD patients in primary care and gastroenterology services [27]. This was combined with data about the odds ratio of complications after delays of <4 months (assumed to represent USC) versus >4 months (assumed to represent delayed diagnosis) [28], to produce an estimate of complications in 46% with delayed diagnosis versus 16% with USC diagnosis (note that uncertainty around each of the separate input parameters was included in the modelling, but 95% CIs for these composite parameters were not calculated). For the purposes of treatment cost allocation in the long-term model, all delayed diagnoses of IBD were assigned a fixed time to diagnosis of 1.34 (1.11-1.69) years reflecting the average time to diagnosis after symptom onset [27].

Each delayed CRC diagnosis was assumed to require an additional 1.9 (1.8-2.0) GP appointments (based on data about average number of GP consultations with relevant symptoms before CRC diagnosis [29]), a colonoscopy (or CTC plus colonoscopy), and have a 29% probability of emergency presentation (calculated by combining Cancer Registry recorded routes to diagnosis [30], a study about emergency diagnosis of CRC [31], and Nottingham data about proportion of diagnoses that were delayed – note that uncertainty around each of the separate input parameters was included in the modelling, but 95% CIs for the composite parameter was not calculated). There was little information available about additional resource requirements for delayed IBD diagnosis, so it was assumed that an equivalent number of additional GP appointments would be required as for CRC delayed diagnosis, in addition to a follow-up colonoscopy. IBD was assumed to not be diagnosed through emergency presentation.

All other individuals who hadn’t been sent for USC investigations were assumed to have a 20% (14.1%-26.6%) probability of having a further FIT following an additional GP appointment (for resource use purposes only, FIT results not modelled) and a 32.5% (19.5%-47.5%) probability of having a follow-up colonoscopy at some point in the future, based on expert elicitation for the previous NICE diagnostic assessment work on symptomatic FIT for triage of suspected CRC patients [12]. For simplicity it was assumed that there was no disease progression prior to this further colonoscopy, but that adenomas present at baseline could be diagnosed and removed through polypectomy if they were detected, based on colonoscopy sensitivity.

## Short-term Model Costs & Utilities

### Short-term Model Costs

Short-term model costs included costs of FIT, GP appointments, COLOFIT, follow-up investigation, endoscopy harms and emergency presentation (Table 4). Costs of disease treatment were not included in the short-term model, but were included in the long-term modelling (see Long-term Markov Model section). All costs were included as per person values, at 2021/22 costs, and where necessary were inflated using the NHS Costs Inflation Index, or if prior to 2015, the Hospital and Community Health Services Index, from the Personal Social Services Research Unit (PSSRU) unit costs publication [32].

Table 4: Per person/episode costs used in the short-term model

| **Cost Parameter** | **Uninflated Cost** | **Inflated Cost** | **Reference & Notes** | **Application** |
| --- | --- | --- | --- | --- |
| FIT | £4.18 | £4.65 | NICE DAG [12]. Includes collection device and test. | All initial and delayed diagnosis FITs. Cost of inadequates in 8% added to cost of each FIT. |
| COLOFIT (annuitised) | £0.01 | £0.01 | Estimate by COLOFIT team. Capital costs. | Incurred each time COLOFIT algorithm run. |
| Phlebotomy | £4.75 | £4.75 | NHS Reference Costs [33]. Taking blood. | Incurred only for COLOFIT algorithm with blood parameters, in the proportion of people who don’t have full blood count data anyway.  . |
| Haemotology | £3.63 | £3.63 | NHS Reference Costs [33]. Full blood count. |  |
| Proportion people without blood test data | 0.09 | NA | Nottingham data |  |
| GP consultation | £42 | £42 | PSSRU [32]. 9.22 minute consultation. | One consultation for each FIT. 1.9 additional consultations for each delayed diagnosis of CRC/IBD |
| Emergency presentation | £296.88 | £296.88 | NHS Reference Costs [33]. Mean emergency medicine costs. | Delayed diagnosis of CRC has 29% probability of emergency presentation. |
| Diagnostic colonoscopy | £919.58 | £919.58 | NHS Reference Costs [33]. Day case costs. | All colonoscopies apart from those where CRC/adenomas diagnosed. |
| Therapeutic colonoscopy | £1,138.88 | £1,138.88 | NHS Reference Costs [33]. Day case costs. | Colonoscopies where CRC or adenomas diagnosed. |
| CTC | £177.95 | £177.95 | NHS Reference Costs [33]. | All CTC examinations. |
| Histopathology & Histology | £32.75 | £32.75 | NHS Reference Costs [33]. | Diagnosis of CRC (x1) or adenomas (x2.3 based on number of adenomas per person). |
| Bowel Perforation | £6,960.59 | £6,960.59 | NHS Reference Costs [33]. Mean major large intestine procedures. | All bowel perforations from colonoscopy or CTC. |
| Bleed requiring hospitalisation | £1,848.34 | £1,848.34 | NHS Reference Costs [33]. Mean GI bleed. | All bowel bleeds from colonoscopy or CTC. |
| FIT faecal immunochemical test; CTC computed tomography colonography; DAG diagnostics advisory guideline; PSSRU personal and social services research unit; GI gastrointestinal; IBD inflammatory bowel disease. | | | | |

Whilst FIT costs are required in both arms of the model, the inclusion of additional FITs in some people not sent for USC could result in different costs being incurred for FIT between arms and so was important to include in the model. The cost of FIT was taken from the previous NICE diagnostic assessment work on symptomatic FIT for triage of suspected CRC patients [12]. They provided the cost of the collection device for two manufacturers and the cost of the test itself for three manufacturers. The mean of these values was taken to obtain a total cost for a FIT of £4.65. Some repeat FITs were required due to inadequate first samples, and the Nottingham data indicated that 8.02% (7.7%-8.3%) more tests were performed than individuals in the sample. Rather than modelling the inadequate samples explicitly, the costs of these additional tests were spread over the cost of each FIT.

The cost of a GP appointment was also included each time an individual had FIT. GP costs were taken from the PSSRU unit costs 2022 publication [32], and were per consultation lasting 9.22 minutes, including direct care with qualification costs. Additional GP appointments were also costed for individuals with delayed diagnosis of CRC or IBD.

The cost of COLOFIT depended upon the algorithm chosen. For COLOFIT either with or without blood results, running the algorithm would be expected to be automated and therefore per person running costs would be negligible, but some capital costs of setting up the required systems and getting regulatory approval would be expected. The COLOFIT team estimated that these costs would be around £100,000. Capital costs were annuitised based on a conservative estimate of a five year lifespan and assuming a 3.5% discount rate, then converted into per person costs assuming roughly 1.88 million people presenting at the GP annually in England with symptoms indicative of CRC. The population figure was calculated based on data indicating that there were 377,163 USC referrals for lower GI cancer in England in 2020-21 [34], and this would represent roughly 20% of the presenting population based on FIT10 positivity in the Nottingham data. This resulted in a total of £0.012 per person.

The main COLOFIT algorithm requires blood results (MCV and platelet count); however, Nottingham data indicated that most people get these currently anyway, as only 9% of patients were missing blood data. The additional cost of a blood test in people with missing data was included as part of the cost of the full COLOFIT algorithm. This was assumed to be equivalent to NHS Reference Costs 2021/22 cost of phlebotomy (taking blood), plus cost of haemotology (full blood count analysis) [33], resulting in a total of £8.38 per person without blood results, or £0.75 when divided across the whole population. These costs come from secondary care, whereas blood tests performed as part of COLOFIT are likely to be taken in primary care; however, it was thought that costs would be similar (e.g. cost of hospital phlebotomy is similar to cost of 5 minutes of primary care nurse time as documented in PSSRU unit costs [32]).

Costs of colonoscopy and CTC were taken from NHS Reference Costs 2021/22 [33]. Diagnostic colonoscopy had lower costs than therapeutic colonoscopy (Table 4). For both types, day case costs were selected over outpatient costs as the vast majority of episodes were recorded as day cases. For CTC, the weighted mean of all Healthcare Resource Groups (HRGs) with the code RD61Z: Colon computerised tomography was selected. Individuals found to have CRC or adenomas during colonoscopy also incurred costs for histopathology and histology (NHS Reference Costs [33]), which for those with adenomas was multiplied by the average number of adenomas found per person with adenomas (2.3 [1.84-2.76] based on screening data [19]). Emergency presentation of CRC was assumed to incur an additional cost over and above the cost of the investigations outlined above. The weighted mean of all categories of emergency medicine apart from dead on arrival and dental was selected to represent this from NHS Reference Costs 2021/22 [33].

Costs were included for bowel perforation and hospital admittance for bleeding as a consequence of endoscopy, both from NHS Reference Costs 2021/22 [33]. The weighted mean of all HRGs with the code FF34: Major large intestine procedures, 19 years and over was chosen to represent bowel perforation, whilst the weighted mean of all HRGs with the code FD03 Gastrointestinal bleed without intervention, with single intervention or multiple intervention was chosen to represent bleeding.

### Short-term Model Utilities

Only utility decrements relating to adverse events from diagnostic procedures were included in the short-term model. This included bowel perforation and bleeding (death was included in the long-term model). It was not possible to find utility decrements relating specifically to endoscopy harms in the literature, and a similar approach was used to that for other health economic modelling of CRC diagnosis [35]. Utility decrements for bleeding were estimated by assuming they would be similar to a major gastrointestinal bleed and used a value of 0.1511 for two weeks [36], equivalent to a utility decrement of 0.00581 (0.00279-0.00883) in the course of a year. Values for perforation were assumed to be the same as for stomach ulcer/abdominal hernia/rupture [9]. The disutility value was reported as 0.118 for one month, equivalent to a utility decrement of 0.00983 (0.00917-0.01058) in the course of a year.

There may be some utility decrement associated with the psychological impacts of being referred via USC or having to wait for a diagnosis; however, no evidence was found to inform the magnitude of such utility decrements, so they were not included in the basecase modelling. Sensitivity analysis was carried out whereby a QALY decrement equivalent to a single day in full quality health was additionally incurred for every colonoscopy undertaken.

# Long-term Markov Model

The long-term model simulates what happens over the remaining life course for each individual in terms of their CRC natural history, diagnosis of CRC beyond the short-term model, treatment costs for CRC and IBD, quality of life and mortality. A diagram is shown in Figure 1B of the main manuscript. The core of the long-term model is the CRC natural history pathway, which is modelled as a Markov model with a set of mutually exclusive CRC-related health states (green boxes). Transitions between health states are modelled as annual transition probabilities. IBD is included as a population characteristic that individuals either have, or don’t have, which influences the direct development of CRC. All individuals have an annual risk of other cause mortality, whilst only those individuals with CRC have a risk of CRC mortality. IBD and CRC health states incur costs and quality of life decrements, which are then combined with those incurred through diagnostics in the short-term model to produce life-time outcomes.

## Long-Term Model Transition Probabilities

### CRC natural history

Cancer natural history transition probabilities cannot be observed and hence are commonly derived for health economic modelling through calibration to external data such as incidence by age and sex, stage distribution and (if available e.g. from diagnostic studies) undiagnosed prevalence. Our previous modelling of screening strategies for CRC involved calibration of natural history parameters to inform transition probabilities for the development and progression of CRC by age and sex [35, 37], and these transition probabilities were recalibrated to updated data for more recent work [38, 39]. In the absence of evidence, it was assumed that CRC natural history transitions in a symptomatic population would be similar to that in the general population, and therefore the updated calibrated transition probabilities were reused in the long-term model (see All Model Parameters and Distributions table). These include estimates of the joint uncertainty around the mean parameters for use in probabilistic sensitivity analysis.

CRC natural history is known to differ in individuals with IBD, who have a much higher risk of CRC than the general population, primarily through a non-adenoma route [40]. Two differences to the CRC natural history transitions were therefore applied to the IBD patients in the model. Firstly, for simplicity it was assumed that all CRC development in people with IBD was through the non-adenoma pathway (i.e. transition probabilities to LR or HR adenomas were set to 0); secondly, an increased risk of CRC through the direct no CRC/adenomas to CRC Stage I pathway was applied both in diagnosed and undiagnosed IBD patients. Data from a population based study was used to inform relative risk of CRC in people with versus without IBD for colon and rectal cancer separately [40], with a weighted average taken based on current incidence data [41], to produce a relative risk of 2.39 (1.74-3.31). As our previous modelling assumed that only 15% of CRC develops through a non-adenoma pathway based on British Society of Gastrology estimates [42], the relative risk was adjusted upward to reflect all additional risk going through the non-adenoma pathway, and applied to the transition from no CRC/adenomas to stage I CRC. No evidence was found to inform whether CRC risk differed between people who had more complications from IBD or spent more time in a severe state compared to those with milder disease, so the same RR was applied to all individuals with IBD in the basecase analysis. Note that it was assumed that no modelled individuals would develop new IBD as part of the long-term model.

### Mortality

Individuals can either die from CRC or from other causes. Both types of mortality were modelled in the same way as in in our previous CRC screening model [35]. CRC mortality was based on Office for National Statistics (ONS) one and five year survival data for people diagnosed between 2013 and 2017 in England, which was given by age, sex and stage [43]. This was combined with survival estimates by sex for each of the 10 years following diagnosis from Cancer Research UK [44], to produce curves for ten year survival by age, sex and stage (see Fig. 15 in [35] for more details). Annual probability of dying due to CRC was calculated from the survival data as follows:

CRC_mort_(age, sex, stage, year)_ = 1 – (CRC_surv_(age, sex, stage, year)_ / CRC_surv_(age, sex, stage, year-1)_)

The same assumptions were used as previously [35]. It was assumed that the probability of dying from CRC beyond ten years post diagnosis was zero. Individuals with undiagnosed CRC for stages I to III were assumed to have no risk of CRC mortality, whilst individuals with undiagnosed stage IV CRC were assumed to have the same risk of CRC mortality as an individual with stage IV CRC in the first year following diagnosis. Of those with undiagnosed stage IV that died from CRC, a proportion were assumed to be diagnosed within the year of death, with the rest remaining undiagnosed, with the proportion in each category based on a previously calibrated parameter (Appendix C).

All-cause mortality by age and sex was obtained from ONS English life tables for 2018-2020 [45]. This includes mortality from CRC and mortality from other causes. Death registration summary statistics for England and Wales (2021) [46] were used to determine the proportion of all registered deaths that were due to CRC. It was assumed that CRC deaths included deaths due to ICD code C18: Malignant neoplasm of colon and ICD codes C19-21: Malignant neoplasm of rectosigmoid junction, rectum and anus. Anal cancer is not included in CRC, but it was not possible to separate this out from rectal cancer in death registration data. Other cause mortality was then calculated by subtracting CRC mortality from all-cause mortality using the following equation:

Oth_Cause_Mort_(age, sex)_ = All_Cause_Mort_(age, sex)_ * (1 – (N_CRC_Deaths_(age, sex)_ / N_All_Deaths_(age, sex)_))

All-cause mortality is higher in people with IBD than people in the general population (relative risk = 0.54 [95% CI 1.44-1.65]) [47]. Whilst some of this increase is due to CRC, most of it is due to other causes. This relative risk was applied uniformly to other cause mortality rates in people with IBD, making the assumption that it did not vary by age or sex. Note that this will slightly over-estimate mortality risks due to some double-counting with CRC. It was assumed that there was no difference in other-cause mortality for people with IBD complications.

### Long-term disease diagnosis

Whilst all CRC in the baseline population was assumed to be diagnosed symptomatically within the short-term model, CRC that developed in the long-term model could be diagnosed through either symptomatic/chance, screening or surveillance pathways. It was also assumed that adenomas could be diagnosed through the screening or surveillance (but not symptomatic/chance) routes. All of these pathways were modelled in a simplified way based on our previous CRC modelling [35, 37, 38, 48]. This was based on the assumption that beyond the short-term model, the COLOFIT population would have the same probability of being diagnosed as the screening/general population.

Symptomatic/chance diagnosis within the long-term model was included as an annual probability of diagnosis for those with CRC, dependent upon CRC stage. Like the natural history transition probabilities, these parameters were calibrated as part of the CRC screening model development [35, 37], and recalibrated to updated data for more recent work [38, 39]. Whilst in practice symptomatic diagnosis will of course differ between model arms, those differences were already fully reflected in the short-term modelling. The purpose here therefore was to reflect long-term differences arising from adenoma detection in the short-term model, and therefore the timing and stage of CRC diagnosis was of primary importance. No resource use or costs of symptomatic diagnosis were included in the long-term modelling, and there was no modelling of symptomatic diagnosis of adenomas or diagnostic processes in people with no CRC/adenomas.

Our previous modelling has simulated the process of CRC screening and surveillance in some detail [35]. This level of detail was unnecessary for modelling COLOFIT, and so was simplified considerably. Our previous CRC screening model was used to derive annual probabilities by age, sex and underlying health state that an individual would be diagnosed with adenomas or CRC through screening or surveillance. Uncertainty around these probabilities was not estimated due to the complexity of obtaining parameter sets based on joint uncertainty estimation. These annual probabilities were then applied to all individuals other than those with a prior diagnosis of CRC. It was assumed that all detected adenomas were removed through polypectomy, whilst all detected CRC was treated. No resource use, costs or harms for screening were included in the long-term modelling as these were unlikely to differ by more than a trivial amount between arms.

## Long-term Model Costs and Utilities

Costs and utilities in the long-term model were discounted by 3.5%

### Long-term Model Costs

Long-term model costs included costs of treatment for CRC and IBD only. All costs were included as per person values, at 2021/22 costs, and where necessary were inflated using the NHS Costs Inflation Index, or if prior to 2015, the Hospital and Community Health Services Index, from the Personal Social Services Research Unit (PSSRU) unit costs publication [32].

CRC treatment costs were taken from a 2016 English costing study that used patient level data to estimate annual costs for each year up to nine years following diagnosis, by age group (18-64 or 65+) and aggregated stage (early and late) [49]. Costs include all healthcare costs incurred by individuals rather than those specifically incurred through CRC treatment, which has the advantage of including indirect costs, but the disadvantage of including unrelated costs that might also be incurred in individuals without cancer. The study does estimate healthcare costs for the three years prior to cancer diagnosis, so cancer-related post-diagnosis healthcare costs were estimated by subtracting the three-years pre-diagnosis costs from the costs for each year post-diagnosis. It was assumed that individuals would not incur further CRC costs beyond nine years post-diagnosis.

IBD costs were taken from a 2015 UK cost of care model for IBD [50], which estimated annual costs for UC and CD separately, whether in remission or relapse. Modelled uncomplicated IBD was assumed to be equivalent to remission for both UC and CD, whereas complicated IBD was assumed to be equivalent to severe disease for UC or any relapse for CD (CD costs were not partitioned by severity). Weighted averages based on prevalence of UC versus CD were used to calculate cost for each health state [51]. As IBD is a chronic condition, costs were assumed to be incurred annually until death.

### Long-term Model Utilities

Long-term modelling of health related quality of life was based on the individual EQ-5D values estimated at baseline (see above), combined with estimates of how these values change over time due to age, symptom improvement, CRC treatment and IBD. Baseline EQ-5D was assigned assuming that all individuals with symptoms had equivalent EQ-5D estimates to patients with bowel/colon complaints (an average of 0.7 [95% CI 0.665-0.731] for someone aged 53, adjusted by age [9]), but it is unlikely that most individuals have symptoms for more than a short period of time unless diagnosed with serious disease. It was therefore assumed that all patients other than those with CRC or IBD would increase their quality of life in the first year to the average in people without bowel/colon complaints (0.83 [95% CI 0.808-0.855] for someone aged 53, adjusted by age) [9]. A multiplier was calculated based on these two values (1.186) and applied in year one for most individuals, or in the year of diagnosis for those with CRC at baseline (to be replaced by CRC-specific utility multipliers as described below). The multiplier was not applied to those with IBD at all, who were assumed to maintain the average quality of life in people with a bowel complaint for the rest of their life if they had uncomplicated disease, with a further decrement incurred if they had complications.

Average quality of life decreases as individuals’ age, due to other conditions not modelled. An annual age decrement (0.00444 [95% CI 0.00409-0.00478]) was calculated based on EQ-5D values by age for the general population [9]. This was applied to all individuals for each year of age.

A review and meta-analysis of CRC utilities was used to estimate utility multipliers for CRC [52] in the same way as used for our previous CRC screening model [35]. The meta-analysis included a linear mixed-effects model for utilities that took into account differences in utility measurements by cancer site, stage, time since surgery, measurement instrument and method of administration. The reference case was chosen for cancer site (CRC) and method of administration (interviewer), whilst EQ-5D was chosen as the measurement instrument. The model also indicated that utility increased after surgery, so the three months post-surgery values were chosen to represent the first year after surgery, and the more than one year post surgery values were chosen to represent subsequent years, up until death from CRC or other causes. Multipliers for stage I-III were calculated as 0.87 (95% CI 0.74-0.99) in year one, rising to 0.92 (95% CI 0.8-1.04) in subsequent years, whereas for stage 4, year one multipliers were 0.68 (95% CI 0.54-0.81) in year one, rising to 0.73 (95% CI 0.61-0.86) in subsequent years. This enabled stage differences to be taken into account post-diagnosis.

Patients with IBD complications were assumed to have lower health-related quality of life than those without complications. A multiplier of 0.73 (95% CI 0.68-0.78) for complicated IBD compared with uncomplicated disease was calculated from EQ-5D scores in active disease and remission for patients with UC and CD, along with their proportions in each group [53].

Individual utility values were modelled annually by first applying the multiplier for symptom improvement if relevant, then applying the total age decrement calculated using the baseline age and EQ-5D for each individual, then applying multipliers due to CRC or complicated IBD (or in a rare few individuals, both CRC and IBD). Utilities were then capped at 1 or -0.594 if necessary, reflecting the upper and lower bounds of the EQ-5D score.

## Integrating Short and Long-term Models

The short-term model simulates diagnosis of all individuals with CRC and IBD at baseline, and some individuals with adenomas at baseline. Whilst diagnostic resource use (and its associated costs and quality of life decrements) are modelled in the short-term model (representing one year of diagnosis), long-term modelling is required to determine whether delays in diagnosis of CRC lead to stage transition, and to simulate the expected timings of initiation of treatment (and hence incurred treatment costs and quality of life decrements) for CRC and IBD.

Several assumptions were made when integrating models. For CRC, it was assumed that there was no progression for those individuals diagnosed through USC. Individuals with delayed diagnosis could transition to a more advanced CRC stage based on their sampled time to diagnosis. In the year of diagnosis, individual transition probabilities were modified to reflect the remaining time to diagnosis by first converting to rates using the following equation:

R = -ln(1-P)

Where R = rate; P = annual transition probability.

These rates were then converted back to probabilities based on remaining time to diagnosis t using the equation:

P = 1-exp^(-Rt)

Where R = rate; t = remaining time to diagnosis in years; P = modified transition probability for time t.

Whilst transition probabilities were modified to take different times to diagnosis into account, this approach was not used for the application of treatment costs and quality of life decrements for CRC, which were applied at year one levels for all live patients from the year of diagnosis, no matter at what point in the year they were diagnosed, for ease of discounting.

For individuals diagnosed with adenomas in the short-term model, it was assumed that all diagnoses (whether through USC or through the additional colonoscopies performed in a random subset of people) occurred in the first modelled year, and that individuals would not progress to a more advanced health state prior to diagnosis. Given that adenoma diagnosis is accompanied by polypectomy, such individuals were assumed to transition to the no CRC/adenomas health state in the first modelled cycle, unless they had died from other cause mortality.

For IBD, all USC diagnoses were assumed to occur in year one, whilst all delayed diagnoses were assumed to occur in year two. Treatment costs and quality of life decrements for IBD were applied in full for all live patients from the year of diagnosis. Increased risks of other cause mortality and CRC were applied to all individuals with IBD, whether or not they had been diagnosed.

All individuals were assumed to be at risk of other cause mortality (and if at CRC stage IV, CRC mortality) from the first modelled year, even if they were allocated to have delayed diagnosis of CRC or IBD. This was important to avoid patients potentially accruing mortality benefits from delayed diagnosis. If a patient did die before their allocated diagnostic time, they did not accrue any costs or quality of life decrements due to treatment in the long-term model; however, their diagnostic outcomes from the short-term model were retained. This ensured that delayed diagnosis did not lead to inappropriate reductions in CRC incidence or diagnostic costs and harms.

# Model Outcomes

The model produced a series of short-term and long-term outcomes as detailed in Table 5. Outcomes were calculated as an average across all modelled individuals to get per person results. Incremental analysis was carried out to compare current care and intervention arms of the model.

Total costs and QALYs were calculated as the sum of short and long-term model results. Cost-effectiveness was estimated from total costs and QALYs using both the incremental cost-effectiveness ratio (ICER = incremental costs / incremental QALYs) and net monetary benefit (NMB = (incremental QALYs * willingness-to-pay threshold) – incremental costs) approaches, assuming a willingness-to-pay threshold of £20,000/QALY.

Table 5: Model outcomes

| **Short-term Model Outcomes** | **Long-term Model Outcomes** |
| --- | --- |
| USC referrals | Long-term diagnosis of CRC and adenomas |
| Diagnosis of CRC, adenomas & IBD at USC and delayed diagnosis | CRC mortality |
| Resource use (e.g. colonoscopy) | Total life years |
| Diagnostic costs | Total QALYs (apart from QoL decrements due to screening harm) |
| Diagnostic harms (costs and QoL decrements) | Treatment costs |
| USC urgent suspected cancer; CRC colorectal cancer; IBD inflammatory bowel disease; QoL quality of life; QALY quality-adjusted life-years. | |

In addition to producing outcomes across the entire population, the model also produced subgroup outcomes by age group (<50, 50-69, 70+) and by sex (male, female). Subgroup outcomes were calculated as an average across all modelled individuals in that subgroup at baseline.

# Model Validation

The implementation of COLOFIT in the model, both to assign health states and predict risk, was tested through validation. The validation compared the ‘actual’ CRC status of the synthetic model populations (assigned through the COLOFIT model), against predicted risk in the synthetic population as assessed by COLOFIT or by FIT score alone. Test characteristics were calculated including sensitivity, specificity and positive predictive value (PPV) (Table 7 & Table 8). These were then compared against the equivalent validation statistics produced by the Nottingham team based on the actual Nottingham data [11]. An investigation of sensitivity and specificity of COLOFIT and FIT10 by age group within the model was also carried out, although the equivalent Nottingham data was not available for comparison.

Table 6: Comparison of test characteristics for FIT and COLOFIT (cox model) by age group within the health economic model for the Nottingham validation population

|  | age <50 | age 50-69 | age 70+ |
| --- | --- | --- | --- |
| COLOFIT 0.64% |  |  |  |
| Positives per 100,000 total population | 2,399 | 5,661 | 11,547 |
| Sensitivity | 0.7913 | 0.8484 | 0.9291 |
| Specificity | 0.9033 | 0.8605 | 0.7159 |
| Area under the receiver operating characteristic curve | 0.9104 | 0.9092 | 0.9066 |
|  |  |  |  |
| FIT 10 |  |  |  |
| Positives per 100,000 total population | 4,243 | 6,027 | 10,259 |
| Sensitivity | 0.8725 | 0.8432 | 0.9109 |
| Specificity | 0.8266 | 0.8515 | 0.7493 |
| Area under the receiver operating characteristic curve | 0.8983 | 0.8928 | 0.8985 |
|  |  |  |  |

The validation results indicate that the health economic model is able to represent the test characteristics relatively closely for the derivation and validation populations. The model is slightly less good at estimating the observed test characteristics for the validation population. This is likely to be because the health economic model uses the relationship between CRC risk and COLOFIT, which was originally developed in the derivation population, to represent the relationship between risk and characteristics such as FIT. As this relationship will differ in the validation population in an unknown way, it is not fully possible for the health economic model to reproduce the observed risk.

Validation results by age indicate that COLOFIT sensitivity increases with age, whilst specificity decreases by age. Area under the receiver operating characteristic curve is overall higher for younger populations than older ones when COLOFIT is used, but this is not the case for FIT.

Table 7: Comparison of test characteristics for FIT and COLOFIT (cox model) within the Nottingham data [11] and within the health economic model for the Nottingham derivation population

|  | PPV (data) | PPV (model) | Sensitivity (data) | Sensitivity (model) | Specificity (data) | Specificity (model) | Positives per 100,000 (data) | Positives per 100,000 (model) |
| --- | --- | --- | --- | --- | --- | --- | --- | --- |
| **COLOFIT 0.64%** | 0.0649 | 0.0648 | 0.9163 | 0.9110 | 0.797 | 0.7979 | 21,383 | 21,282 |
| **COLOFIT 1%** | 0.0792 | 0.0764 | 0.8906 | 0.8904 | 0.8408 | 0.8346 | 17,033 | 17,637 |
| **COLOFIT 2%** | 0.1032 | 0.0981 | 0.8493 | 0.8458 | 0.8864 | 0.8805 | 12,472 | 11,769 |
| **COLOFIT 3%** | 0.1239 | 0.1152 | 0.8178 | 0.8043 | 0.911 | 0.9050 | 10,001 | 9,354 |
| **FIT 10** | 0.0607 | 0.0606 | 0.8988 | 0.9081 | 0.7861 | 0.7834 | 21,919 | 21,329 |
| **FIT 40** | 0.1172 | 0.1132 | 0.778 | 0.7787 | 0.9098 | 0.9062 | 10,637 | 9,234 |
| PPV positive predictive value; FIT faecal immunochemical test | | | | | | | | |

Table 8: Comparison of test characteristics for FIT and COLOFIT (cox model) within the Nottingham data [11] and within the health economic model for the Nottingham validation population

|  | PPV (data) | PPV (model) | Sensitivity (data) | Sensitivity (model) | Specificity (data) | Specificity (model) | Positives per 100,000 (data) | Positives per 100,000 (model) |
| --- | --- | --- | --- | --- | --- | --- | --- | --- |
| **COLOFIT 0.64%** | 0.0611 | 0.0559 | 0.923 | 0.8948 | 0.822 | 0.8181 | 18,681 | 19,036 |
| **COLOFIT 1%** | 0.0759 | 0.0660 | 0.912 | 0.8720 | 0.861 | 0.8513 | 14,856 | 15,733 |
| **COLOFIT 2%** | 0.0976 | 0.0837 | 0.874 | 0.8198 | 0.899 | 0.8919 | 11,080 | 11,660 |
| **COLOFIT 3%** | 0.115 | 0.0978 | 0.83 | 0.7712 | 0.92 | 0.9143 | 8,917 | 9,323 |
| **FIT 10** | 0.0492 | 0.0460 | 0.9305 | 0.9072 | 0.7747 | 0.7733 | 23,406 | 23,477 |
| **FIT 40** | 0.0971 | 0.0902 | 0.837 | 0.7679 | 0.903 | 0.9067 | 10,654 | 10,137 |
| PPV positive predictive value; FIT faecal immunochemical test | | | | | | | | |

# All Model Parameters & Distributions

| **Parameter Name** | **Mean** | **Lower 95% CI** | **Upper 95% CI** | **Distribution** | **Source** |
| --- | --- | --- | --- | --- | --- |
| Transition probability: Normal to LR Adenoma Age 35 Male | 6.42E-04 | NA | NA | Correlated | Calibrated CRC natural history model parameter sets [35, 37]. |
| Transition probability: Normal to LR Adenoma Age 45 Male | 2.02E-02 | NA | NA | Correlated |  |
| Transition probability: Normal to LR Adenoma Age 55 Male | 1.89E-02 | NA | NA | Correlated |  |
| Transition probability: Normal to LR Adenoma Age 65 Male | 6.22E-04 | NA | NA | Correlated |  |
| Transition probability: Normal to LR Adenoma Age 75 Male | 2.90E-03 | NA | NA | Correlated |  |
| Transition probability: Normal to LR Adenoma Age 85 Male | 2.34E-04 | NA | NA | Correlated |  |
| Transition probability: Normal to LR Adenoma Age 35 Female | 3.60E-04 | NA | NA | Correlated |  |
| Transition probability: Normal to LR Adenoma Age 45 Female | 1.05E-02 | NA | NA | Correlated |  |
| Transition probability: Normal to LR Adenoma Age 55 Female | 1.02E-02 | NA | NA | Correlated |  |
| Transition probability: Normal to LR Adenoma Age 65 Female | 3.74E-03 | NA | NA | Correlated |  |
| Transition probability: Normal to LR Adenoma Age 75 Female | 4.13E-04 | NA | NA | Correlated |  |
| Transition probability: Normal to LR Adenoma Age 85 Female | 5.20E-03 | NA | NA | Correlated |  |
| Transition probability: LR to HR Adenoma Age 35 Male | 2.82E-02 | NA | NA | Correlated |  |
| Transition probability: LR to HR Adenoma Age 45 Male | 3.13E-02 | NA | NA | Correlated |  |
| Transition probability: LR to HR Adenoma Age 55 Male | 2.06E-02 | NA | NA | Correlated |  |
| Transition probability: LR to HR Adenoma Age 65 Male | 1.21E-02 | NA | NA | Correlated |  |
| Transition probability: LR to HR Adenoma Age 75 Male | 1.55E-02 | NA | NA | Correlated |  |
| Transition probability: LR to HR Adenoma Age 85 Male | 9.27E-03 | NA | NA | Correlated |  |
| Transition probability: LR to HR Adenoma Age 35 Female | 1.75E-02 | NA | NA | Correlated |  |
| Transition probability: LR to HR Adenoma Age 45 Female | 2.85E-02 | NA | NA | Correlated |  |
| Transition probability: LR to HR Adenoma Age 55 Female | 1.45E-02 | NA | NA | Correlated |  |
| Transition probability: LR to HR Adenoma Age 65 Female | 1.44E-02 | NA | NA | Correlated |  |
| Transition probability: LR to HR Adenoma Age 75 Female | 1.99E-02 | NA | NA | Correlated |  |
| Transition probability: LR to HR Adenoma Age 85 Female | 1.14E-02 | NA | NA | Correlated |  |
| Transition probability: HR to Cancer Age 35 Male | 9.2E-03 | NA | NA | Correlated |  |
| Transition probability: HR to Cancer Age 45 Male | 1.6E-02 | NA | NA | Correlated |  |
| Transition probability: HR to Cancer Age 55 Male | 1.8E-02 | NA | NA | Correlated |  |
| Transition probability: HR to Cancer Age 65 Male | 2.8E-02 | NA | NA | Correlated |  |
| Transition probability: HR to Cancer Age 75 Male | 5.0E-02 | NA | NA | Correlated |  |
| Transition probability: HR to Cancer Age 85 Male | 3.5E-02 | NA | NA | Correlated |  |
| Transition probability: HR to Cancer Age 35 Female | 4.7E-03 | NA | NA | Correlated |  |
| Transition probability: HR to Cancer Age 45 Female | 2.1E-02 | NA | NA | Correlated |  |
| Transition probability: HR to Cancer Age 55 Female | 2.7E-02 | NA | NA | Correlated |  |
| Transition probability: HR to Cancer Age 65 Female | 3.6E-02 | NA | NA | Correlated |  |
| Transition probability: HR to Cancer Age 75 Female | 6.5E-02 | NA | NA | Correlated |  |
| Transition probability: HR to Cancer Age 85 Female | 5.3E-02 | NA | NA | Correlated |  |
| Transition probability: Normal to Cancer at age 15 Males | 0 | NA | NA | Correlated |  |
| Transition probability: Normal to Cancer at age 101 Males | 3.2E-04 | NA | NA | Correlated |  |
| Transition probability: Normal to Cancer at age 15 Females | 0 | NA | NA | Correlated |  |
| Transition probability: Normal to Cancer at age 101 Females | 4.5E-04 | NA | NA | Correlated |  |
| Transition probability: Undiagnosed CRC: Stage I to II | 2.93E-01 | NA | NA | Correlated |  |
| Transition probability: Undiagnosed CRC: Stage II to III | 5.54E-01 | NA | NA | Correlated |  |
| Transition probability: Undiagnosed CRC: Stage III to IV | 3.50E-01 | NA | NA | Correlated |  |
| Proportion CRC Stage IV deaths undiagnosed as function of age >75 | 0.04 | NA | NA | Correlated |  |
| Symptomatic presentation rate with CRC Stage I | 2.03E-02 | NA | NA | Correlated |  |
| Symptomatic presentation rate with CRC Stage II | 1.43E-01 | NA | NA | Correlated |  |
| Symptomatic presentation rate with CRC Stage III | 2.74E-01 | NA | NA | Correlated |  |
| Symptomatic presentation rate with CRC Stage IV | 2.50E-01 | NA | NA | Correlated |  |
| Symptomatic presentation annual decrement in people aged over 75 | 3.61E-02 | NA | NA | Correlated |  |
| Average number of adenomas present in patient with at least one | 2.3 | 2.3 | 2.3 | Lognormal | Rutter et al 2014 [19] |
| COLOFIT cox survival algorithm: baseline hazard | -0.659 | 1.504 | 1.833 | Constant | Crooks et al 2024 [11] |
| COLOFIT cox survival algorithm: age/100 cubed term | 1.669 | -15.648 | -12.257 | Normal |  |
| COLOFIT cox survival algorithm: ln age/100 * age/100 cubed term | -13.944 | -2.120 | -1.897 | Normal |  |
| COLOFIT cox survival algorithm: FIT/100 square rooted term | -1.997 | -0.288 | -0.236 | Normal |  |
| COLOFIT cox survival algorithm: ln FIT/100 * FIT/100 square rooted term | -0.266 | 0.842 | 1.001 | Normal |  |
| COLOFIT cox survival algorithm: ln Platelets/100 term | 0.921 | -4.605 | -3.507 | Normal |  |
| COLOFIT cox survival algorithm: MCV term | -3.901 | 0.399 | 0.513 | Normal |  |
| COLOFIT cox survival algorithm: male term | 0.454 | 1.504 | 1.833 | Normal |  |
| COLOFIT logistic algorithm: baseline hazard | 0.122 | 0 | 0 | Constant |  |
| COLOFIT logistic algorithm: age/100 cubed term | 1.963 | 1.790 | 2.139 | Normal |  |
| COLOFIT logistic algorithm: ln age/100 * age/100 cubed term | -15.093 | -16.895 | -13.346 | Normal |  |
| COLOFIT logistic algorithm: FIT/100 square rooted term | -2.193 | -2.303 | -2.120 | Normal |  |
| COLOFIT logistic algorithm: ln FIT/100 * FIT/100 square rooted term | -0.316 | -0.342 | -0.288 | Normal |  |
| COLOFIT logistic algorithm: ln Platelets/100 term | 1.072 | 0.990 | 1.163 | Normal |  |
| COLOFIT logistic algorithm: MCV term | -4.732 | -5.116 | -4.343 | Normal |  |
| COLOFIT logistic algorithm: male term | 0.512 | 0.399 | 0.513 | Normal |  |
| Relative risk CRC in people with IBD vs those without | 15.96 | 11.61 | 22.07 | Lognormal | Bernstein et al 2001 [40] |
| Prevalence CRC in baseline symptomatic population | 0.015 | 0.014 | 0.016 | Beta | Crooks et al 2024 [11] |
| Prevalence CRC in baseline symptomatic population: age <50 | 0.004 | 0.003 | 0.006 | Beta |  |
| Prevalence CRC in baseline symptomatic population: age 50-70 | 0.010 | 0.008 | 0.012 | Beta |  |
| Prevalence CRC in baseline symptomatic population: age 70+ | 0.024 | 0.022 | 0.027 | Beta |  |
| Prevalence adenoma >5mm in FIT>=10 population | 0.115 | 0.108 | 0.122 | Beta | Nottingham population (C. Crooks personal communication) |
| Prevalence HR adenomas in baseline symptomatic population | 0.043 | 0.039 | 0.047 | Beta | Dsouza et al 2021a [6] |
| Prevalence LR adenomas in baseline symptomatic population | 0.236 | 0.228 | 0.245 | Beta |  |
| Prevalence IBD in baseline symptomatic population | 0.043 | 0.040 | 0.048 | Beta |  |
| Prevalence IBD in baseline symptomatic population: age <50 | 0.079 | 0.064 | 0.095 | Beta | Dsouza et al 2021b [8] |
| Prevalence IBD in baseline symptomatic population: age 50+ | 0.039 | 0.035 | 0.043 | Beta |  |
| Percentage baseline CRC stage 1 | 0.213 | 0.179 | 0.249 | Beta | Nottingham population (C. Crooks personal communication) |
| Percentage baseline CRC stage 2 | 0.157 | 0.127 | 0.189 | Beta |  |
| Percentage baseline CRC stage 3 | 0.313 | 0.274 | 0.353 | Beta |  |
| Percentage baseline CRC stage 4 | 0.187 | 0.155 | 0.222 | Beta |  |
| Percentage baseline CRC missing stage | 0.130 | 0.103 | 0.160 | Beta |  |
| Colonoscopy uptake USC | 0.9808 | 0.978 | 0.983 | Beta | Dsouza et al 2021a [6] |
| CTC uptake USC | 0.9672 | 0.959 | 0.975 | Beta | Stephenson et al 2018 [16] |
| Proportion CTC of all referrals USC | 0.117 | 0.090 | 0.148 | Beta | NICE DG30 2017 [12] |
| FIT repeat test rate (inadequates) | 0.0802 | 0.077 | 0.083 | Beta | Nottingham data |
| Colonoscopy repeat test rate USC (inadequates) | 0.101 | 0.095 | 0.106 | Beta | Dsouza et al 2021a [6] |
| Colonoscopy (with polypectomy) perforation rate | 0.00091 | 0.00061 | 0.00128 | Beta | Rutter et al 2014 [19] |
| Colonoscopy (without polypectomy) perforation rate | 0.00031 | 0.00014 | 0.00054 | Beta |  |
| CTC perforation rate | 0.0008 | 0.000 | 0.0030 | Beta | Bellini et al 2014 [20] |
| Colonoscopy probability of hospitalisation due to bleeding | 0.0005 | 0.0003 | 0.0009 | Beta | Gavin et al 2013 [21] |
| Colonoscopy probability of death | 0.000031 | 0.00001 | 0.00008 | Beta | Tomaszweski et al 2021 [22] |
| CTC probability of death | 0 | 0 | 0 | Constant | Bellini et al 2014 [20] |
| Time to CRC delayed diagnosis parameter lognormal mean | -1.386294 | -1.43063 | -1.341959 | Normal | Ahri et al 2020 [24] |
| Time to CRC delayed diagnosis parameter lognormal distribution | 1.0624432 | 1.018108 | 1.1067786 | Normal |  |
| Probability previous GP attendance for CRC symptoms with emergency CRC diagnosis | 0.186 | 0.151 | 0.223 | Beta | Renzi et al 2016 [31] |
| Proportion CRC diagnoses that are emergency | 0.215 | 0.211 | 0.220 | Beta | English cancer registry data [30] |
| Proportion CRC diagnoses that are GP referral | 0.561 | 0.555 | 0.566 | Beta |  |
| Average no. additional GP appointments if delayed diagnosis of CRC | 1.919 | 1.821 | 2.017 | Normal | Lyratzopoulos et al 2013 [29] |
| Time to IBD delayed diagnosis years | 1.344 | 1.107 | 1.692 | Lognormal | Walker et al 2020 [27] |
| Probability diagnosed within 6 months of symptom onset for IBD | 0.60 | 0.543 | 0.653 | Beta |  |
| Proportion of IBD patients who have complications | 0.30 | 0.249 | 0.352 | Beta |  |
| OR IBD complications (diagnosed >4 m compared to <4 m) | 2.884 | 0.542 | 16.994 | Lognormal | Nguyen et al 2017 [28] |
| Probability FIT/COLOFIT -ve patients offered colonoscopy later | 0.325 | 0.191 | 0.475 | Beta | NICE DG30 2017 [12] |
| Probability FIT/COLOFIT -ve patients offered FIT later | 0.2000 | 0.141 | 0.266 | Beta |  |
| Colonoscopy sensitivity for LR adenomas | 0.765 | 0.733 | 0.796 | Beta | Van Rijn et al 2006 [13] |
| Colonoscopy sensitivity for HR adenomas | 0.925 | 0.894 | 0.952 | Beta | Martin-Lopez at al 2014 [14] |
| Colonoscopy sensitivity for CRC | 0.965 | 0.917 | 0.993 | Beta | Than et al 2015 [15] |
| Colonoscopy sensitivity for IBD | 1 | 1 | 1 | Constant | Assumption due to nature of test. |
| Colonoscopy specificity CRC | 1 | 1 | 1 | Constant |  |
| Colonoscopy specificity for IBD | 1 | 1 | 1 | Constant |  |
| CTC sensitivity for LR adenomas | 0.627 | 0.381 | 1.018 | Beta | Based on detection rates relative to colonoscopy from Atkin et al 2013 [17] |
| CTC sensitivity for HR adenomas | 0.759 | 0.465 | 1.218 | Beta |  |
| CTC sensitivity for CRC | 0.945 | 0.577 | 1.509 | Beta |  |
| CTC sensitivity for IBD | 0.84 | 0.791 | 0.891 | Beta | Horsthuis et al 2008 [18] |
| CTC false positive rate | 0.197 | 0.164 | 0.232 | Beta | Atkin et al 2013 [17] |
| PPV of FIT>=150 for IBD in age < 50 | 0.427 | 0.330 | 0.527 | Beta | Dsouza et al 2021b [8]. Estimated by subtracting PPV for advanced neoplasia from PPV for all serious bowel disease. |
| PPV of FIT>=10 for IBD in age < 50 | 0.309 | 0.246 | 0.376 | Beta |  |
| NPV of FIT>=10 for IBD in age < 50 | 0.970 | 0.949 | 0.985 | Beta |  |
| PPV of FIT>=150 for IBD in age >= 50 | 0.177 | 0.149 | 0.207 | Beta |  |
| PPV of FIT>=10 for IBD in age >= 50 | 0.112 | 0.097 | 0.128 | Beta |  |
| NPV of FIT>=10 for IBD in age >= 50 | 0.979 | 0.974 | 0.984 | Beta |  |
| Baseline EQ5D mean value bowel symptoms | 0.70 | 0.665 | 0.731 | Normal | Ara & Brazier 2011 [9] |
| Baseline EQ5D mean value no bowel symptoms | 0.83 | 0.808 | 0.855 | Normal |  |
| Baseline EQ5D standard deviation | 0.0168 | 0 | 0 | Constant |  |
| Utility decrement age mean | 0.00444 | 0.00409 | 0.00478 | Normal |  |
| Utility decrement age standard deviation | 0.00018 | 0 | 0 | Constant |  |
| Utility multiplier CRC Yr1 Stage I to III | 0.87 | 0.74 | 0.99 | Normal | Djalalov et al 2014 [52] |
| Utility multiplier CRC Yr1 Stage IV | 0.68 | 0.54 | 0.81 | Normal |  |
| Utility multiplier CRC Yr2+ Stage I to III | 0.92 | 0.8 | 1.04 | Normal |  |
| Utility multiplier CRC Yr2+ Stage IV | 0.73 | 0.61 | 0.86 | Normal |  |
| Utility multiplier IBD complications | 0.728 | 0.676 | 0.780 | Normal | Stark et al 2010 [53] |
| Utility decrement screening harm perforation | -0.00983 | -0.01058 | -0.00917 | Normal | Ara & Brazier 2011 [9] |
| Utility decrement screening harm bleeding requiring hospitalisation | -0.00581 | -0.00883 | -0.00279 | Normal | Dorian et al 2014 [36] |
| Other cause mortality relative risk in people with IBD vs without | 1.54 | 1.44 | 1.65 | Lognormal | Card et al 2003 [47] |
| CRC treatment cost: stage I & II, Age <64, Year 1 | £17,709.39 | £14,409 | £21,345 | Gamma | Laudicella et al 2016. [49] Excess costs compared with 3 years before cancer diagnosis. |
| CRC treatment cost: stage I & II, Age <64, Year 2 | £4,155.79 | £3,381 | £5,009 | Gamma |  |
| CRC treatment cost: stage I & II, Age <64, Year 3 | £3,448.91 | £2,806 | £4,157 | Gamma |  |
| CRC treatment cost: stage I & II, Age <64, Year 4 | £2,663.75 | £2,167 | £3,211 | Gamma |  |
| CRC treatment cost: stage I & II, Age <64, Year 5 | £2,396.42 | £1,950 | £2,888 | Gamma |  |
| CRC treatment cost: stage I & II, Age <64, Year 6 | £1,638.96 | £1,334 | £1,975 | Gamma |  |
| CRC treatment cost: stage I & II, Age <64, Year 7 | £1,703.98 | £1,386 | £2,054 | Gamma |  |
| CRC treatment cost: stage I & II, Age <64, Year 8 | £1,561.89 | £1,271 | £1,883 | Gamma |  |
| CRC treatment cost: stage I & II, Age <64, Year 9 | £1,346.33 | £1,095 | £1,623 | Gamma |  |
| CRC treatment cost: stage I & II, Age 65+, Year 1 | £16,548.52 | £13,465 | £19,946 | Gamma |  |
| CRC treatment cost: stage I & II, Age 65+, Year 2 | £3,811.39 | £3,101 | £4,594 | Gamma |  |
| CRC treatment cost: stage I & II, Age 65+, Year 3 | £3,106.91 | £2,528 | £3,745 | Gamma |  |
| CRC treatment cost: stage I & II, Age 65+, Year 4 | £2,584.28 | £2,103 | £3,115 | Gamma |  |
| CRC treatment cost: stage I & II, Age 65+, Year 5 | £2,622.81 | £2,134 | £3,161 | Gamma |  |
| CRC treatment cost: stage I & II, Age 65+, Year 6 | £2,650.51 | £2,157 | £3,195 | Gamma |  |
| CRC treatment cost: stage I & II, Age 65+, Year 7 | £2,408.46 | £1,960 | £2,903 | Gamma |  |
| CRC treatment cost: stage I & II, Age 65+, Year 8 | £2,669.78 | £2,172 | £3,218 | Gamma |  |
| CRC treatment cost: stage I & II, Age 65+, Year 9 | £2,229.03 | £1,814 | £2,687 | Gamma |  |
| CRC treatment cost: stage III & IV, Age <64, Year 1 | £22,868.31 | £18,607 | £27,563 | Gamma |  |
| CRC treatment cost: stage III & IV, Age <64, Year 2 | £7,490.30 | £6,094 | £9,028 | Gamma |  |
| CRC treatment cost: stage III & IV, Age <64, Year 3 | £5,120.38 | £4,166 | £6,172 | Gamma |  |
| CRC treatment cost: stage III & IV, Age <64, Year 4 | £4,182.29 | £3,403 | £5,041 | Gamma |  |
| CRC treatment cost: stage III & IV, Age <64, Year 5 | £2,985.28 | £2,429 | £3,598 | Gamma |  |
| CRC treatment cost: stage III & IV, Age <64, Year 6 | £2,498.78 | £2,033 | £3,012 | Gamma |  |
| CRC treatment cost: stage III & IV, Age <64, Year 7 | £2,911.83 | £2,369 | £3,510 | Gamma |  |
| CRC treatment cost: stage III & IV, Age <64, Year 8 | £2,232.64 | £1,817 | £2,691 | Gamma |  |
| CRC treatment cost: stage III & IV, Age <64, Year 9 | £1,535.39 | £1,249 | £1,851 | Gamma |  |
| CRC treatment cost: stage III & IV, Age 65+, Year 1 | £18,059.82 | £14,694 | £21,767 | Gamma |  |
| CRC treatment cost: stage III & IV, Age 65+, Year 2 | £5,694.80 | £4,634 | £6,864 | Gamma |  |
| CRC treatment cost: stage III & IV, Age 65+, Year 3 | £4,396.64 | £3,577 | £5,299 | Gamma |  |
| CRC treatment cost: stage III & IV, Age 65+, Year 4 | £3,442.89 | £2,801 | £4,150 | Gamma |  |
| CRC treatment cost: stage III & IV, Age 65+, Year 5 | £3,221.31 | £2,621 | £3,883 | Gamma |  |
| CRC treatment cost: stage III & IV, Age 65+, Year 6 | £3,058.74 | £2,489 | £3,687 | Gamma |  |
| CRC treatment cost: stage III & IV, Age 65+, Year 7 | £1,955.67 | £1,591 | £2,357 | Gamma |  |
| CRC treatment cost: stage III & IV, Age 65+, Year 8 | £2,539.72 | £2,066 | £3,061 | Gamma |  |
| CRC treatment cost: stage III & IV, Age 65+, Year 9 | £1,974.94 | £1,607 | £2,380 | Gamma |  |
| IBD annual treatment cost no complications | £1,735.36 | £1,412 | £2,092 | Gamma | Ghosh et al 2015 [50] |
| IBD annual treatment cost with complications | £10,662.58 | £8,675 | £12,851 | Gamma |  |
| Cost of symptomatic FIT collection device (all invited) | £1.70 | £1.38 | £2.05 | Gamma | NICE DG30 2017 [12] |
| Cost of symptomatic FIT processing (only responders) | £2.95 | £2.40 | £3.56 | Gamma |  |
| Capital costs COLOFIT | £100,000 | £81,364 | £120,529 | Gamma | Assumption |
| Cost of GP consultation | £42.00 | £34 | £51 | Gamma | PSSRU Costs 2022 [54] |
| Proportion of people missing blood tests | 0.09 | 0.087 | 0.093 | Beta | Nottingham population (C. Crooks personal communication) |
| Extra costs of blood tests required for COLOFIT | £8.38 | £6.82 | £10.10 | Gamma | NHS Reference Costs 2022 [33] |
| Cost of A&E attendance | £296.88 | £242 | £358 | Gamma |  |
| Cost of colonoscopy (without polypectomy) | £919.58 | £748 | £1,108 | Gamma |  |
| Cost of colonoscopy (with polypectomy) | £1,138.88 | £927 | £1,373 | Gamma |  |
| Cost of CTC | £177.95 | £145 | £214 | Gamma |  |
| Cost of treating bowel perforation (major surgery) | £6,960.59 | £5,663 | £8,390 | Gamma |  |
| Cost of admittance for bleeding (overnight stay on medical ward) | £1,848.34 | £1,504 | £2,228 | Gamma |  |
| Biopsy cost | £32.75 | £27 | £39 | Gamma |  |

# References

1. *DG30 Quantitative Faecal immunochemical tests to guide referral for colorectal cancer in primary care*. Diagnostics Guidance, NICE 2017 [cited 2022 24th April]; Available from: <https://www.nice.org.uk/guidance/dg30>.

2. *NG151 Colorectal Cancer*. NICE Guidance 2021 [cited 2022 24th April]; Available from: <https://www.nice.org.uk/guidance/ng151>.

3. Chapman, C., et al., *Service evaluation of faecal immunochemical testing and anaemia for risk stratification in the 2-week-wait pathway for colorectal cancer.* BJS Open, 2019. **3**(3): p. 395-402.

4. Bailey, J.A., et al., *Sociodemographic variations in the uptake of Faecal Immunochemical Tests (FIT) in a primary care symptomatic pathway for colorectal cancer.* In Press, 2023.

5. Robinson, A.G., et al., *Is cancer stage data missing completely at random? A report from a large population-based cohort of non-small cell lung cancer.* Frontiers in Oncology, 2023. **13**.

6. D'Souza, N., et al., *Faecal immunochemical test is superior to symptoms in predicting pathology in patients with suspected colorectal cancer symptoms referred on a 2WW pathway: a diagnostic accuracy study.* Gut, 2021. **70**(6): p. 1130-1138.

7. Wong, M.C.S., et al., *Global Prevalence of Colorectal Neoplasia: A Systematic Review and Meta-Analysis.* Clin Gastroenterol Hepatol, 2020. **18**(3): p. 553-561 e10.

8. D'Souza, N., et al., *Finding the needle in the haystack: the diagnostic accuracy of the faecal immunochemical test for colorectal cancer in younger symptomatic patients.* Colorectal Dis, 2021. **23**(10): p. 2539-2549.

9. Ara, R. and J. Brazier, *Using health state utility values from the general population to approximate baselines in decision analytic models when condition-specific data are not available.* Value in Health, 2011. **2011**(4): p. 539-45.

10. Bailey, S.E.R., et al., *Diagnostic performance of a faecal immunochemical test for patients with low-risk symptoms of colorectal cancer in primary care: an evaluation in the South West of England.* Br J Cancer, 2021. **124**(7): p. 1231-1236.

11. Crooks, C.J., et al., *COLOFIT: Development and internal-external validation of models using age, sex, faecal immunochemical and blood tests to optimise diagnosis of colorectal cancer in symptomatic patients.* medRxiv preprint, 2024.

12. Westwood, M., et al., *Faecal immunochemical tests to triage patients with lower abdominal symptoms for suspected colorectal cancer referrals in primary care: a systematic review and cost-effectiveness analysis.* Health Technol Assess, 2017. **21**(33): p. 1-234.

13. van Rijn, J.C., et al., *Polyp miss rate determined by tandem colonoscopy: a systematic review.* Am J Gastroenterol, 2006. **101**(2): p. 343-50.

14. Martin-Lopez, J.E., et al., *Comparison of the accuracy of CT colonography and colonoscopy in the diagnosis of colorectal cancer.* Colorectal Dis, 2014. **16**(3): p. O82-9.

15. Than, M., et al., *Diagnostic miss rate for colorectal cancer: an audit.* Annals of Gastroenterology, 2015. **28**(1): p. 94-98.

16. Stephenson, J.A., et al., *Straight-to-test faecal tagging CT colonography for exclusion of colon cancer in symptomatic patients under the English 2-week-wait cancer investigation pathway: a service review.* Clin Radiol, 2018. **73**(9): p. 836 e1-836 e7.

17. Atkin, W., et al., *Computed tomographic colonography versus colonoscopy for investigation of patients with symptoms suggestive of colorectal cancer (SIGGAR): a multicentre randomised trial.* The Lancet, 2013. **381**(9873): p. 1194-1202.

18. Horsthuis, K., et al., *Inflammatory bowel disease diagnosed with US, MR, scintigraphy, and CT: meta-analysis of prospective studies.* Radiology, 2008. **247**(1): p. 64-79.

19. Rutter, M., et al., *Risk factors for adverse events related to polypectomy in the English Bowel Cancer Screening Programme.* Endoscopy, 2014. **46**(02): p. 90-97.

20. Bellini, D., et al., *Perforation rate in CT colonography: a systematic review of the literature and meta-analysis.* Eur Radiol, 2014. **24**(7): p. 1487-96.

21. Gavin, D.R., et al., *The national colonoscopy audit: a nationwide assessment of the quality and safety of colonoscopy in the UK.* Gut, 2013. **62**(2): p. 242-9.

22. Tomaszewski, M., et al., *Risks associated with colonoscopy in a population-based colon screening program: an observational cohort study.* CMAJ Open, 2021. **9**(4): p. E940-E947.

23. Monahan, K.J., et al., *Faecal immunochemical testing (FIT) in patients with signs or symptoms of suspected colorectal cancer (CRC): a joint guideline from the Association of Coloproctology of Great Britain and Ireland (ACPGBI) and the British Society of Gastroenterology (BSG).* Gut, 2022.

24. Arhi, C.S., et al., *Delays in referral from primary care worsen survival for patients with colorectal cancer: a retrospective cohort study.* Br J Gen Pract, 2020. **70**(696): p. e463-e471.

25. *NG130 Ulcerative Colitis: Management*. NICE Guideline 2019 [cited 2022 1st September]; Available from: <https://www.nice.org.uk/guidance/ng130>.

26. *NG129 Crohn's Disease: Management*. NICE Guideline 2019 [cited 2022 1st September]; Available from: <https://www.nice.org.uk/guidance/ng129>.

27. Walker, G.J., et al., *Quality improvement project identifies factors associated with delay in IBD diagnosis.* Aliment Pharmacol Ther, 2020. **52**(3): p. 471-480.

28. Nguyen, V.Q., et al., *Impact of Diagnostic Delay and Associated Factors on Clinical Outcomes in a U.S. Inflammatory Bowel Disease Cohort.* Inflamm Bowel Dis, 2017. **23**(10): p. 1825-1831.

29. Lyratzopoulos, G., et al., *Measures of promptness of cancer diagnosis in primary care: secondary analysis of national audit data on patients with 18 common and rarer cancers.* Br J Cancer, 2013. **108**(3): p. 686-90.

30. *Early Diagnosis Data Hub*. 2022 [cited 2024 31 July]; Available from: <https://crukcancerintelligence.shinyapps.io/EarlyDiagnosis/>.

31. Renzi, C., et al., *Do colorectal cancer patients diagnosed as an emergency differ from non-emergency patients in their consultation patterns and symptoms? A longitudinal data-linkage study in England.* Br J Cancer, 2016. **115**(7): p. 866-75.

32. Jones, K.C., et al. *Unit Costs of Health and Social Care 2022 Manual*. Technical report. 2023 [cited 2023 1st April]; Available from: <https://www.pssru.ac.uk/unitcostsreport/>.

33. *2021/22 National Cost Collection data*. National Cost Collection for the NHS 2022 [cited 2023 1st April]; Available from: <https://www.england.nhs.uk/costing-in-the-nhs/national-cost-collection/>.

34. Coward, A., K. Moon, and P. McDonnell. *Waiting Times for Suspected and Diagnosed Cancer Patients: 2020-21 Annual Report*. 2021 [cited 2023 1st July]; Available from: <https://www.england.nhs.uk/statistics/wp-content/uploads/sites/2/2021/07/Cancer-Waiting-Times-Annual-Report-202021-Final.pdf>.

35. Thomas, C., O. Mandrik, and S. Whyte, *Development of the Microsimulation Model in Cancer of the Bowel (MiMiC-Bowel), an Individual Patient Simulation Model for Investigation of the Cost effectiveness of Personalised Screening and Surveillance Strategies*, in *HEDS Discussion Paper Series*. 2020.

36. Dorian, P., et al., *Cost-effectiveness of apixaban vs. current standard of care for stroke prevention in patients with atrial fibrillation.* Eur Heart J, 2014. **35**(28): p. 1897-906.

37. Mandrik, O., et al., *Calibration and Validation of the Microsimulation Model in Cancer of the Bowel (MiMiC-Bowel), an Individual Patient Simulation Model for Investigation of the Cost-effectiveness of Personalised Screening*. 2021.

38. Mandrik, O., J. Chilcott, and C. Thomas, *Modelling the impact of the coronavirus pandemic on bowel cancer screening outcomes in England: A decision analysis to prepare for future screening disruption.* Prev Med, 2022. **160**: p. 107076.

39. Thomas, C., O. Mandrik, and S. Whyte, *Modelling cost-effective strategies for minimising socioeconomic inequalities in colorectal cancer screening outcomes in England.* Prev Med, 2022. **162**: p. 107131.

40. Bernstein, C.N., et al., *Cancer risk in patients with inflammatory bowel disease: a population-based study.* Cancer, 2001. **91**(4): p. 854-62.

41. *Bowel Cancer: Stages, types and grades*. Cancer Research UK 2021 [cited 2022 24th April]; Available from: <https://www.cancerresearchuk.org/about-cancer/bowel-cancer/stages-types-and-grades>.

42. East, J.E., et al., *British Society of Gastroenterology position statement on serrated polyps in the colon and rectum.* Gut, 2017. **66**(7): p. 1181-1196.

43. *Adult Cancer Survival Tables: One and Five Year net survival for adults diagnosed between 2013 and 2017, England*. [cited 2019 20th October]; Available from: <https://www.ons.gov.uk/peoplepopulationandcommunity/healthandsocialcare/conditionsanddiseases/datasets/cancersurvivalratescancersurvivalinenglandadultsdiagnosed>.

44. *Bowel Cancer Survival Statistics*. [cited 2019 12th May]; Available from: <http://www.cancerresearchuk.org/health-professional/cancer-statistics/statistics-by-cancer-type/bowel-cancer/survival#heading-One>.

45. *National Life Tables: England*. Office for National Statistics 2021 [cited 2022 1st September]; Available from: <https://www.ons.gov.uk/peoplepopulationandcommunity/birthsdeathsandmarriages/lifeexpectancies/datasets/nationallifetablesenglandreferencetables>.

46. *Mortality statistics - underlying cause, sex and age*. Office for National Statistics; nomis official census and labour market statistics 2021 [cited 2022 1st September]; Available from: <https://www.nomisweb.co.uk/query/construct/summary.asp?mode=construct&version=0&dataset=161>.

47. Card, T., R. Hubbard, and R.F. Logan, *Mortality in Inflammatory Bowel Disease: A Population-Based Cohort Study.* Gastroenterology, 2003. **125**: p. 1583-1590.

48. Thomas, C., O. Mandrik, and S. Whyte, *Modelling cost-effective strategies for minimising health inequalities in colorectal cancer screening.* Preventive Medicine, Submitted.

49. Laudicella, M., et al., *Cost of care for cancer patients in England: evidence from population-based patient-level data.* Br J Cancer, 2016. **114**(11): p. 1286-92.

50. Ghosh, N. and P. Premchand, *A UK cost of care model for inflammatory bowel disease.* Frontline Gastroenterol, 2015. **6**(3): p. 169-174.

51. Pasvol, T.J., et al., *Incidence and prevalence of inflammatory bowel disease in UK primary care: a population-based cohort study.* BMJ Open, 2020. **10**(7): p. e036584.

52. Djalalov, S., et al., *A Review and Meta-analysis of Colorectal Cancer Utilities.* Med Decis Making, 2014. **34**(6): p. 809-18.

53. Stark, R.G., et al., *Validity, reliability, and responsiveness of the EQ-5D in inflammatory bowel disease in Germany.* Inflamm Bowel Dis, 2010. **16**(1): p. 42-51.

54. *Unit Costs of Health and Social Care 2021*. Personal Social Services Research Unit 2022 [cited 2022 1st September]; Available from: <https://www.pssru.ac.uk/project-pages/unit-costs/unit-costs-of-health-and-social-care-2021/>.
