# Supplementary material for: Cost-effectiveness of FIT and a FIT-based model to optimise symptomatic diagnosis of colorectal cancer: health economic modelling for the COLOFIT project
Source: BMJ Public Health. 2025 Jun 24;3(1):e002089. doi: 10.1136/bmjph-2024-002089 (PMC12198831; doi:10.1136/bmjph-2024-002089)
Supplement: online supplemental file 2 [file bmjph-3-1-s002.docx]

# CHEERS 2022 Checklist

Please note that reported locations refer to the clean copy of the manuscript.

| **Topic** | **No.** | **Item** | **Location where item is reported** |
| --- | --- | --- | --- |
| **Title** |  |  |  |
|  | 1 | Identify the study as an economic evaluation and specify the interventions being compared. | Page 1, line 1 |
| **Abstract** |  |  |  |
|  | 2 | Provide a structured summary that highlights context, key methods, results, and alternative analyses. | Page 2 |
| **Introduction** |  |  |  |
| **Background and objectives** | 3 | Give the context for the study, the study question, and its practical relevance for decision making in policy or practice. | Pages 4-5 |
| **Methods** |  |  |  |
| **Health economic analysis plan** | 4 | Indicate whether a health economic analysis plan was developed and where available. | A HEAP was not developed but conceptual modelling was carried out as mentioned on page 6, line 9 & supplementary technical methods file section 1. |
| **Study population** | 5 | Describe characteristics of the study population (such as age range, demographics, socioeconomic, or clinical characteristics). | Paragraph starting on page 6, line 19 & supplementary technical methods file section 3. |
| **Setting and location** | 6 | Provide relevant contextual information that may influence findings. | Page 5, line 17-18 |
| **Comparators** | 7 | Describe the interventions or strategies being compared and why chosen. | Paragraph starting on page 9, line 19 |
| **Perspective** | 8 | State the perspective(s) adopted by the study and why chosen. | Page 6, line 5 |
| **Time horizon** | 9 | State the time horizon for the study and why appropriate. | Page 6, line 8-9 |
| **Discount rate** | 10 | Report the discount rate(s) and reason chosen. | Page 9, line 17-18 |
| **Selection of outcomes** | 11 | Describe what outcomes were used as the measure(s) of benefit(s) and harm(s). | Page 9, line 13 |
| **Measurement of outcomes** | 12 | Describe how outcomes used to capture benefit(s) and harm(s) were measured. | Paragraph starting on page 8, line 11 & supplementary technical methods file sections 4-5 |
| **Valuation of outcomes** | 13 | Describe the population and methods used to measure and value outcomes. | Utilities sections of supplementary technical methods file sections 4-5 |
| **Measurement and valuation of resources and costs** | 14 | Describe how costs were valued. | Two paragraphs starting on page 7, line 19 & Costs sections of supplementary technical methods file sections 4-5 |
| **Currency, price date, and conversion** | 15 | Report the dates of the estimated resource quantities and unit costs, plus the currency and year of conversion. | Page 9, line 17 & Costs sections of supplementary technical methods file sections 4-5 |
| **Rationale and description of model** | 16 | If modelling is used, describe in detail and why used. Report if the model is publicly available and where it can be accessed. | Pages 6-10, Figure 1 & supplementary technical methods file. Data sharing statement on page 19, lines 8-11. |
| **Analytics and assumptions** | 17 | Describe any methods for analysing or statistically transforming data, any extrapolation methods, and approaches for validating any model used. | Synthetic population construction reported in paragraph starting page 6, line 19, and supplementary technical methods file section 3. Model validation reported on page 9, lines 8-12 & supplementary technical methods file section 7 |
| **Characterising heterogeneity** | 18 | Describe any methods used for estimating how the results of the study vary for subgroups. | Page 9, lines 16-17 & supplementary technical methods file |
| **Characterising distributional effects** | 19 | Describe how impacts are distributed across different individuals or adjustments made to reflect priority populations. | Supplementary technical methods file describes differences in input parameters by age and sex subgroups. |
| **Characterising uncertainty** | 20 | Describe methods to characterise any sources of uncertainty in the analysis. | Probabilistic sensitivity analysis and scenario analyses to investigate structural uncertainty described on page 10 from line 4 & PSA parameters & distributions table is in supplementary technical methods file section 8. |
| **Approach to engagement with patients and others affected by the study** | 21 | Describe any approaches to engage patients or service recipients, the general public, communities, or stakeholders (such as clinicians or payers) in the design of the study. | Supplementary technical methods file section 1. |
| **Results** |  |  |  |
| **Study parameters** | 22 | Report all analytic inputs (such as values, ranges, references) including uncertainty or distributional assumptions. | Supplementary technical methods file – throughout but parameter table found specifically in section 8 |
| **Summary of main results** | 23 | Report the mean values for the main categories of costs and outcomes of interest and summarise them in the most appropriate overall measure. | Paragraph 1, page 11, Table 1 |
| **Effect of uncertainty** | 24 | Describe how uncertainty about analytic judgments, inputs, or projections affect findings. Report the effect of choice of discount rate and time horizon, if applicable. | Throughout results pages 11-13, Table 1, Table 2, Figure 2, Supplementary Tables and Figures. |
| **Effect of engagement with patients and others affected by the study** | 25 | Report on any difference patient/service recipient, general public, community, or stakeholder involvement made to the approach or findings of the study | No differences to the study approach or findings were made due to PPI or stakeholder input. |
| **Discussion** |  |  |  |
| **Study findings, limitations, generalisability, and current knowledge** | 26 | Report key findings, limitations, ethical or equity considerations not captured, and how these could affect patients, policy, or practice. | Pages 14-17 |
| **Other relevant information** |  |  |  |
| **Source of funding** | 27 | Describe how the study was funded and any role of the funder in the identification, design, conduct, and reporting of the analysis | Page 18, lines 2-5 |
| **Conflicts of interest** | 28 | Report authors conflicts of interest according to journal or International Committee of Medical Journal Editors requirements. | Page 18, line 7 |

*From:* Husereau D, Drummond M, Augustovski F, et al. Consolidated Health Economic Evaluation Reporting Standards 2022 (CHEERS 2022) Explanation and Elaboration: A Report of the ISPOR CHEERS II Good Practices Task Force. Value Health 2022;25. <doi:10.1016/j.jval.2021.10.008>
