# Supplementary material for: Cost-effectiveness of FIT and a FIT-based model to optimise symptomatic diagnosis of colorectal cancer: health economic modelling for the COLOFIT project
Source: BMJ Public Health. 2025 Jun 24;3(1):e002089. doi: 10.1136/bmjph-2024-002089 (PMC12198831; doi:10.1136/bmjph-2024-002089)

Supplementary Table S1: Subgroup results by age and sex obtained through probabilistic sensitivity analysis comparing USC referral based on different FIT and COLOFIT thresholds. NMB and costs are presented per person in that subgroup, all other outcomes are presented per 100,000 people in that subgroup.

| **Analysis** | **Outcome (incremental)** | **Subgroup** | | | | | |
| --- | --- | --- | --- | --- | --- | --- | --- |
|  |  | **All** | **Male** | **Female** | **Age <50** | **Age 50-70** | **Age 70+** |
| FIT 10 versus sending all to USC | NMB | £460 | £450 | £468 | £476 | £485 | £426 |
|  | Costs | -£469 | -£459 | -£476 | -£483 | -£496 | -£432 |
|  | QALYs | -43 | -48 | -38 | -35 | -57 | -32 |
|  | LYs | -59 | -67 | -53 | -44 | -79 | -48 |
|  | USC CRC Diag | -139 | -171 | -112 | -55 | -134 | -192 |
|  | USC Referrals | -79,757 | -78,150 | -81,054 | -82,298 | -84,507 | -73,520 |
|  | Prob. CE | 100% | 100% | 100% | 100% | 100% | 100% |
| COLOFIT 0.64% versus sending all to USC | NMB | £466 | £445 | £483 | £519 | £491 | £410 |
|  | Costs | -£475 | -£454 | -£492 | -£531 | -£503 | -£415 |
|  | QALYs | -47 | -48 | -46 | -60 | -59 | -28 |
|  | LYs | -65 | -66 | -63 | -75 | -81 | -42 |
|  | USC CRC Diag | -136 | -155 | -121 | -85 | -136 | -165 |
|  | USC Referrals | -81,008 | -77,442 | -83,885 | -90,496 | -85,708 | -70,809 |
|  | Prob. CE | 100% | 100% | 100% | 100% | 100% | 100% |
| COLOFIT 0.64% versus FIT 10 | NMB | £6 | -£5 | £14 | £42 | £6 | -£16 |
|  | Costs | -£7 | £5 | -£16 | -£47 | -£6 | £17 |
|  | QALYs | -4 | 0 | -8 | -25 | -1 | 4 |
|  | LYs | -5 | 1 | -10 | -31 | -2 | 6 |
|  | USC CRC Diag | 3 | 16 | -8 | -30 | -2 | 27 |
|  | USC Referrals | -1,251 | 708 | -2,831 | -8,197 | -1,201 | 2,711 |
|  | Prob. CE | 98% | 2% | 100% | 100% | 96% | 0% |
| FIT40 versus sending all to USC | NMB | £513 | £504 | £519 | £532 | £523 | £491 |
|  | Costs | -£529 | -£523 | -£534 | -£547 | -£543 | -£506 |
|  | QALYs | -84 | -97 | -73 | -76 | -99 | -73 |
|  | LYs | -116 | -134 | -102 | -95 | -136 | -109 |
|  | USC CRC Diag | -284 | -359 | -223 | -109 | -228 | -441 |
|  | USC Referrals | -90,022 | -88,976 | -90,867 | -93,108 | -92,347 | -85,906 |
|  | Prob. CE | 100% | 100% | 100% | 100% | 100% | 100% |
| COLOFIT 3% versus sending all to USC | NMB | £514 | £499 | £525 | £544 | £526 | £483 |
|  | Costs | -£532 | -£519 | -£543 | -£567 | -£549 | -£496 |
|  | QALYs | -93 | -97 | -90 | -115 | -111 | -63 |
|  | LYs | -128 | -132 | -124 | -144 | -152 | -94 |
|  | USC CRC Diag | -277 | -312 | -248 | -169 | -247 | -368 |
|  | USC Referrals | -90,663 | -88,337 | -92,539 | -96,629 | -93,471 | -84,398 |
|  | Prob. CE | 100% | 100% | 100% | 100% | 100% | 100% |
| COLOFIT 3% versus FIT 40 | NMB | £1 | -£5 | £6 | £12 | £3 | -£8 |
|  | Costs | -£3 | £5 | -£9 | -£20 | -£6 | £10 |
|  | QALYs | -10 | 0 | -18 | -40 | -12 | 10 |
|  | LYs | -11 | 2 | -22 | -49 | -16 | 15 |
|  | USC CRC Diag | 7 | 47 | -26 | -60 | -19 | 72 |
|  | USC Referrals | -640 | 638 | -1,672 | -3,521 | -1,124 | 1,509 |
|  | Prob. CE | 66% | 10% | 88% | 87% | 79% | 1% |
| USC urgent suspected cancer; FIT faecal immunochemical test; COLOFIT algorithm developed for COLOFIT project; NMB net monetary benefit (calculated based on a threshold of £20,000 per QALY); QALY quality-adjusted life-year; CRC colorectal cancer; Diag diagnoses; Prob. CE probability cost-effective. | | | | | | | |

Supplementary Figure S1: Graphs comparing FIT and COLOFIT at different thresholds against referring all urgently, in subgroups by sex and age for A) incremental net monetary benefit per person; B) incremental QALYs per person; C) incremental costs per person; D) total USC referrals per 100,000 people and E) incremental USC CRC diagnoses per 100,000 people.


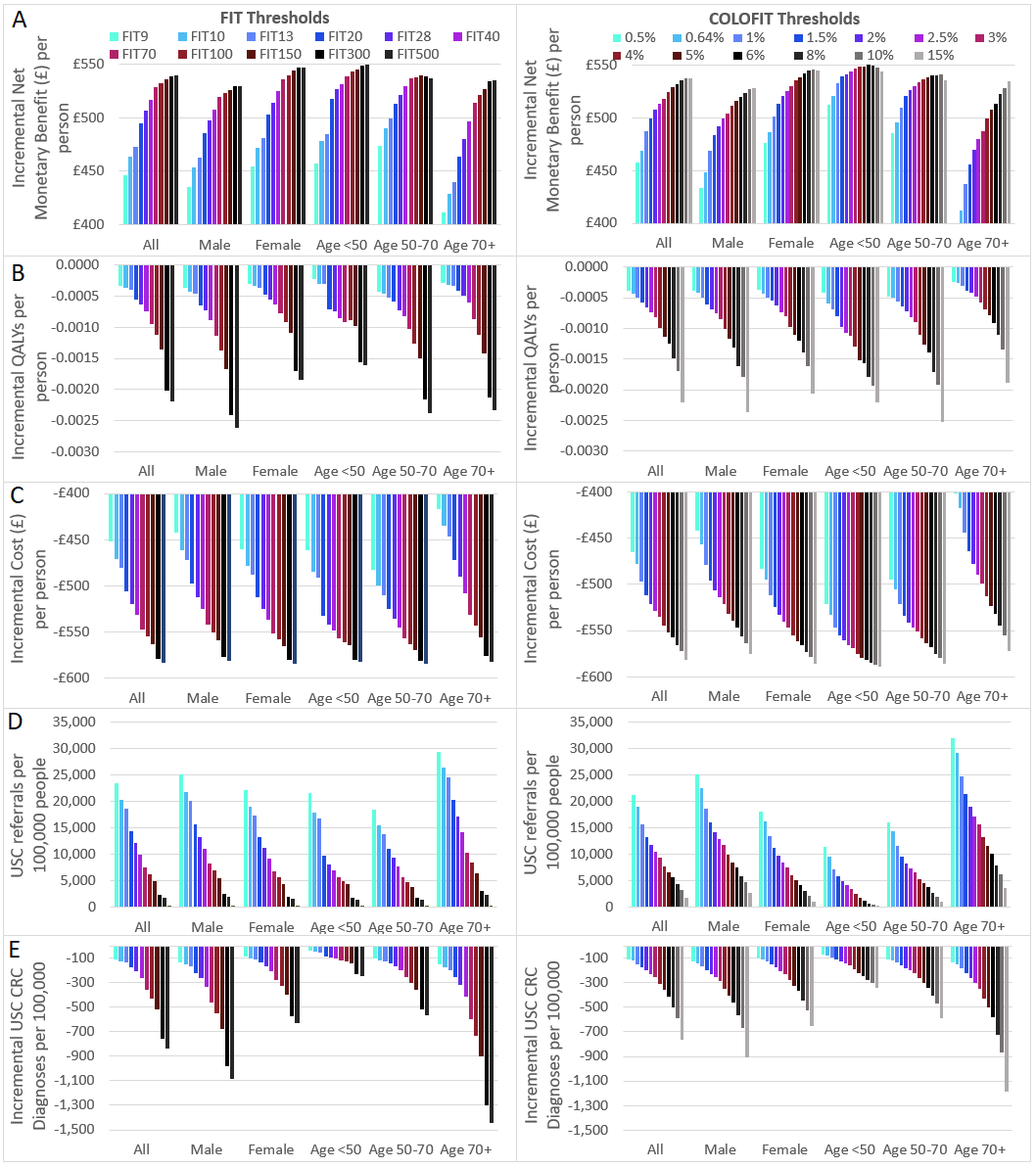


Supplementary Table S2: Comparison of incremental outcomes for basecase and scenario analyses comparing FIT and COLOFIT thresholds against referring all urgently. Note that basecase results vary slightly from those presented in Table S1 as these are deterministic analyses. NMB and costs are presented per person in that subgroup, all other outcomes are presented per 100,000 people in that subgroup.

| **Scenario** | **Outcome (incremental)** | **0.64% Risk Threshold** | | | **3% Risk Threshold** | | |
| --- | --- | --- | --- | --- | --- | --- | --- |
|  |  | **FIT 10 vs refer all** | **COLOFIT 0.64% vs refer all** | **COLOFIT 0.64% vs FIT 10** | **FIT 40 vs refer all** | **COLOFIT 3% vs refer all** | **COLOFIT 3% vs FIT 40** |
| Basecase | NMB | £464 | £469 | £6 | £517 | £519 | £2 |
|  | Costs | -£471 | -£478 | -£7 | -£532 | -£535 | -£3 |
|  | QALYs | -37 | -43 | -6 | -75 | -82 | -8 |
|  | USC CRC Diag | -124 | -121 | 2 | -262 | -255 | 7 |
|  | USC Referrals | -79,728 | -81,012 | -1,284 | -89,993 | -90,643 | -649 |
| 1a) Include adenomas in the model | NMB | -£405 | -£420 | -£16 | -£478 | -£492 | -£14 |
|  | Costs | -£357 | -£358 | -£1 | -£402 | -£401 | £2 |
|  | QALYs | -3809 | -3892 | -82 | -4402 | -4465 | -62 |
|  | USC CRC Diag | -124 | -121 | 2 | -262 | -255 | 7 |
|  | USC Referrals | -79,728 | -81,012 | -1,284 | -89,993 | -90,643 | -649 |
| 1b) Include adenomas & IBD in the model | NMB | -£1,016 | -£1,267 | -£251 | -£1,506 | -£1,637 | -£131 |
|  | Costs | £77 | £241 | £164 | £328 | £411 | £83 |
|  | QALYs | -4692 | -5126 | -434 | -5893 | -6130 | -236 |
|  | USC CRC Diag | -124 | -121 | 2 | -262 | -255 | 7 |
|  | USC Referrals | -79,728 | -81,012 | -1,284 | -89,993 | -90,643 | -649 |
| 2) Pop. based on Nottingham derivation cohort | NMB | £461* | £457* | -£4* | £515* | £512* | -£3* |
|  | Costs | -£468 | -£464 | £4 | -£531 | -£528 | £3 |
|  | QALYs | -38 | -37 | 1 | -79 | -80 | -2 |
|  | USC CRC Diag | -143 | -128 | 14 | -315 | -273 | 42 |
|  | USC Referrals | -79,333 | -78,742 | 591 | -89,788 | -89,498 | 290 |
| 3) Use logistic COLOFIT algorithm | NMB | £463 | £474 | £10 | £517 | £521 | £3 |
|  | Costs | -£471 | -£481 | -£10 | -£532 | -£537 | -£5 |
|  | QALYs | -37 | -36 | 1 | -73 | -84 | -11 |
|  | USC CRC Diag | -121 | -118 | 3 | -252 | -249 | 3 |
|  | USC Referrals | -79,743 | -81,597 | -1,854 | -90,012 | -91,038 | -1,026 |
| 4) Addition of colonoscopy harm | NMB | £491 | £497 | £6 | £548 | £550 | £2 |
|  | Costs | -£471 | -£478 | -£7 | -£532 | -£535 | -£3 |
|  | QALYs | 100 | 96 | -4 | 79 | 73 | -7 |
|  | USC CRC Diag | -124 | -121 | 2 | -262 | -255 | 7 |
|  | USC Referrals | -79,728 | -81,012 | -1,284 | -89,993 | -90,643 | -649 |
| 5a) Doubled diagnostic delay | NMB | £459 | £464 | £5 | £509 | £509 | £0 |
|  | Costs | -£473 | -£480 | -£7 | -£536 | -£539 | -£3 |
|  | QALYs | -71 | -82 | -11 | -137 | -150 | -13 |
|  | USC CRC Diag | -124 | -121 | 2 | -262 | -255 | 7 |
|  | USC Referrals | -79,728 | -81,012 | -1,284 | -89,993 | -90,643 | -649 |
| 5b) Faster stage transitions for delayed diagnoses | NMB | £441 | £444 | £3 | £472 | £469 | -£3 |
|  | Costs | -£474 | -£481 | -£7 | -£538 | -£541 | -£3 |
|  | QALYs | -165 | -183 | -18 | -328 | -359 | -31 |
|  | USC CRC Diag | -124 | -121 | 2 | -262 | -255 | 7 |
|  | USC Referrals | -79,728 | -81,012 | -1,284 | -89,993 | -90,643 | -649 |
| 5c) Combination of 5a and 5b | NMB | £432 | £435 | £3 | £457 | £451 | -£6 |
|  | Costs | -£477 | -£484 | -£7 | -£544 | -£547 | -£3 |
|  | QALYs | -226 | -247 | -21 | -439 | -482 | -43 |
|  | USC CRC Diag | -124 | -121 | 2 | -262 | -255 | 7 |
|  | USC Referrals | -79,728 | -81,012 | -1,284 | -89,993 | -90,643 | -649 |
| 6a) Discount rates increased to 5% | NMB | £465 | £471 | £6 | £519 | £521 | £2 |
|  | Costs | -£471 | -£478 | -£7 | -£532 | -£535 | -£3 |
|  | QALYs | -32 | -36 | -5 | -63 | -69 | -6 |
|  | USC CRC Diag | -124 | -121 | 2 | -262 | -255 | 7 |
|  | USC Referrals | £465 | £471 | £6 | £519 | £521 | £2 |
| 6b) Discount rates reduced to 1.5% | NMB | £462 | £467 | £5 | £513 | £514 | £1 |
|  | Costs | -£471 | -£478 | -£7 | -£532 | -£535 | -£3 |
|  | QALYs | -47 | -57 | -9 | -97 | -108 | -11 |
|  | USC CRC Diag | -124 | -121 | 2 | -262 | -255 | 7 |
|  | USC Referrals | -79,728 | -81,012 | -1,284 | -89,993 | -90,643 | -649 |
| 7) COLOFIT costs increased | NMB | £464 | £462 | -£2 | £517 | £511 | -£6 |
|  | Costs | -£471 | -£470 | £1 | -£532 | -£527 | £4 |
|  | QALYs | -37 | -43 | -6 | -75 | -82 | -8 |
|  | USC CRC Diag | -124 | -121 | 2 | -262 | -255 | 7 |
|  | USC Referrals | -79,728 | -81,012 | -1,284 | -89,993 | -90,643 | -649 |
| IBD inflammatory bowel disease; COLOFIT algorithm developed for COLOFIT project; USC urgent suspected cancer; FIT faecal immunochemical test; NMB net monetary benefit (calculated based on a threshold of £20,000 per QALY); QALY quality-adjusted life-year; CRC colorectal cancer; Diag diagnoses; *For this population the FIT:COLOFIT comparison does not result in equal numbers of CRC diagnosis; if these are equalised COLOFIT is more cost-effective than FIT at both thresholds. | | | | | | | |

Supplementary Figure S2a: Plots for basecase and scenario analyses comparing incremental net monetary benefit, costs and QALYs against USC CRC diagnoses for different thresholds of FIT and COLOFIT compared to sending all urgently.


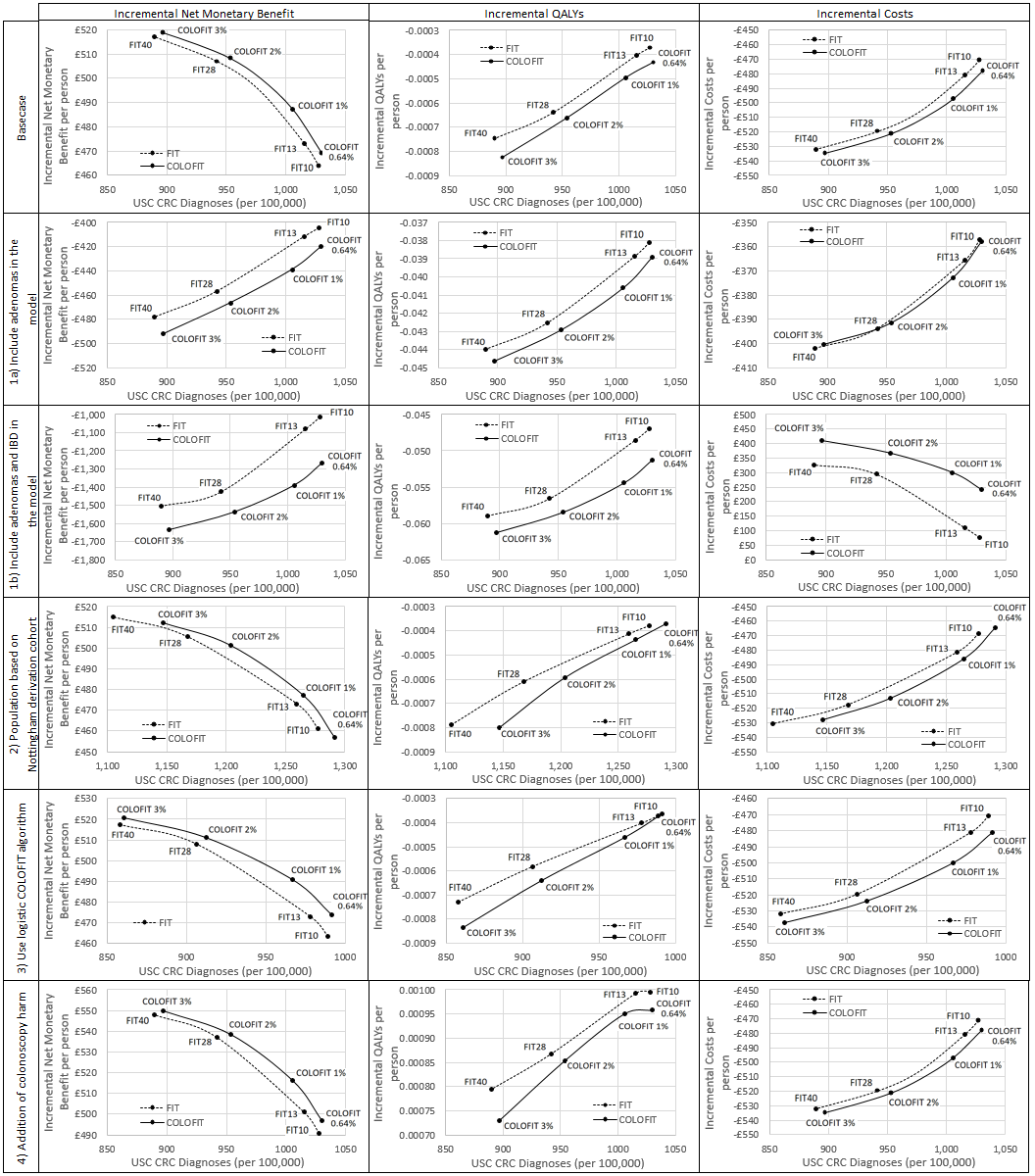


Supplementary Figure S2b: Plots for scenario analyses comparing incremental net monetary benefit, costs and QALYs against USC CRC diagnoses for different thresholds of FIT and COLOFIT compared to sending all urgently.


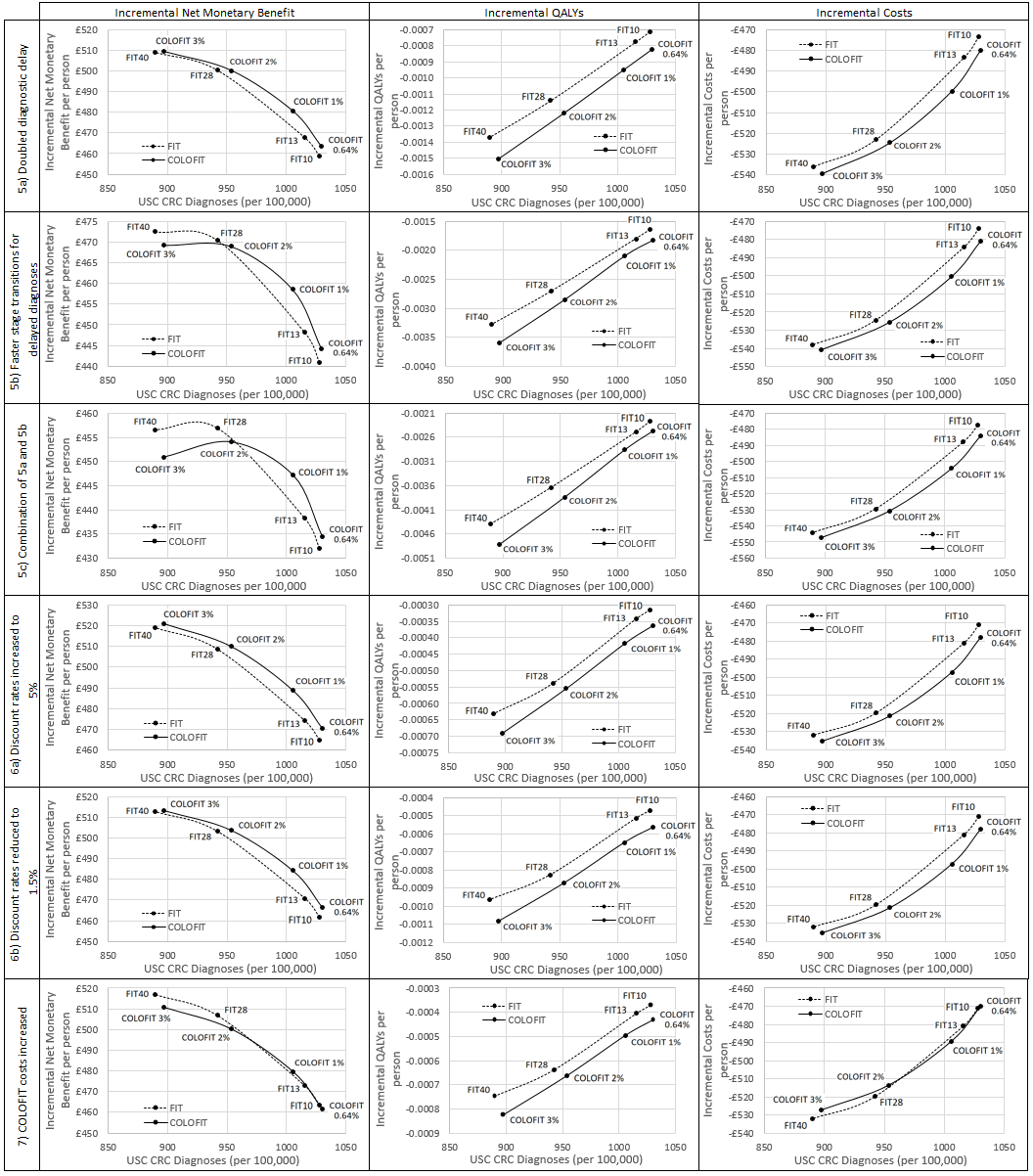

Supplement: online supplemental file 3 [file bmjph-3-1-s003.docx]
